# Cytotoxic Triterpenes from Argan Pulp (*Argania spinosa*): Isolation, In Vitro Evaluation on Cancer Cell Lines, and In Silico Studies

**DOI:** 10.3390/molecules31173011

**Published:** 2026-08-27

**Authors:** Asma El Kaourat, Badr Eddine Kartah, Mohamed El Fadili, Noura Bentarhlia, Hamza Tachallait, Yassine Jaouhari, Matteo Bordiga, Zineb Ourradi, Younes Zaid, Zoubida Charrouf, Hanae El Monfalouti

**Affiliations:** 1 Laboratory of Plant Chemistry, Organic and Bioorganic Synthesis, Faculty of Sciences, Mohammed V University in Rabat, Agdal, Rabat 10000, Morocco; asma.elkaourat@gmail.com (A.E.K.); b.kartah@um5r.ac.ma (B.E.K.); noura.bentarhlia@um5r.ac.ma (N.B.); zcharrouf@yahoo.fr (Z.C.); h.elmonfalouti@um5r.ac.ma (H.E.M.); 2 Laboratoire d’Ingénierie, Modélisation et Analyse des Systèmes (LIMAS Laboratory), Chemistry Department, Faculty of Sciences Dhar El Mahraz, Sidi Mohamed Ben Abdellah University, Fez 30000, Morocco; mohamed.elfadili@usmba.ac.ma; 3 Department of Chemical and Biochemical Sciences—Green Process Engineering (CBS-GPE), Mohammed VI Polytechnic University (UM6P), Ben Guerir 43150, Morocco; hamza.tachallait@um6p.ma; 4 Department of Pharmaceutical Science, Università degli Studi del Piemonte Orientale “A. Avogadro”, Largo Donegani 2, 28100 Novara, Italy; matteo.bordiga@uniupo.it; 5 Materials, Nanotechnologies and Environment Laboratory, Department of Biology, Faculty of Sciences, Mohammed V University in Rabat, Rabat 10000, Morocco; ourradizineb@gmail.com (Z.O.); y.zaid@um5r.ac.ma (Y.Z.)

**Keywords:** argan pulp, pentacyclic triterpenes, erythrodiol, amyrin, sustainability, by-product

## Abstract

This study focuses on the isolation, characterization, and evaluation of cytotoxicity of triterpenes extracted from argan pulp. The unsaponifiable lipids extracted from argan pulp are separated into five fractions, two of which are triterpene fractions F2 (composed of α-amyrin, β-amyrin, lupeol, ψ-taraxasterol, taraxasterol) and F4 (erythrodiol). The extraction process utilized solvent-based methods followed by purification through column chromatography. Structural elucidation was carried out using GC-MS and NMR techniques. The cytotoxic activity of the fractions was evaluated against HepG2, MCF-7, and HeLa cell lines. Fraction F4 exhibited IC_50_ values of 45.58, 71.24, and 98.80 µg/mL, respectively, while fraction F2 showed IC_50_ values of 122.6, 167.8, and 212.7 µg/mL. Annexin V–FITC/PI flow-cytometry analysis was performed on HepG2 cancer cells and THLE-2 non-tumor cells treated with F4. The results showed a concentration-dependent increase in apoptosis in HepG2 cells, with minimal necrosis. To complement the experimental results, computational analyses were performed to evaluate the pharmacokinetic properties. The triterpenes showed favorable drug-like characteristics and low predicted toxicity. Molecular docking revealed strong interactions with key cancer-related targets, including BCL-2, estrogen receptor α, and HPV16 E6.

## 1. Introduction

Conventional cancer treatments, such as chemotherapy, remain central to cancer care but are often limited by systemic toxicity and the frequent emergence of therapeutic resistance [1,2]. These adverse effects compromise patients’ quality of life and reduce long-term treatment efficacy. Consequently, complementary therapeutic strategies are increasingly being explored, including the use of bioactive natural compounds [3,4], which are attractive due to their potential to selectively target tumor cells. Among these compounds, triterpenes widely distributed throughout the plant kingdom stand out for their promising anticancer properties, warranting further investigation as alternative or adjuvant therapies [5,6]. Their low toxicity profiles and ability to sensitize tumor cells to conventional treatments make them particularly appealing candidates for combination therapies [7,8].

In addition to their anticancer potential, triterpenes exhibit notable anti-inflammatory and antioxidant activities, further supporting their suitability as more targeted and less toxic anticancer agents [9,10]. Current research indicates that triterpenes modulate several key processes involved in cancer progression [11]. These include inhibition of cell proliferation, induction of apoptosis (programmed cell death), and suppression of cancer cell migration and invasion [6]. Mechanistically, triterpenes exert their effects by blocking specific signaling pathways that are frequently overactivated in many cancer types and contribute to tumor growth, survival, and resistance to therapy [12]. By inhibiting these pathways, triterpenes limit cancer cell proliferation and viability, rendering tumor cells more susceptible to treatment. This targeted mode of action offers a dual advantage by reducing tumor progression while minimizing damage to healthy cells, thereby potentially improving patients’ quality of life.

Oncology research relies on a variety of cancer cell models to evaluate the effects of triterpenes across different tumor types. This approach is essential for understanding how these compounds interact with the distinct molecular characteristics of each cancer. In liver cancer research, HepG2 cells derived from human hepatocellular carcinoma represent a well-established in vitro model for investigating the efficacy of triterpenes in inhibiting key survival pathways, including PI3K/Akt and MAPK/ERK [13].These pathways are frequently activated in liver cancer and are closely associated with rapid cell proliferation and resistance to therapy. Targeting these pathways with triterpenes may therefore reduce liver cancer cell growth and support the development of more specific therapeutic strategies.

Similarly, in breast cancer research, MCF-7 cells derived from estrogen-dependent breast carcinoma are widely used to assess the effects of triterpenes on hormone-regulated pathways involved in cancer cell proliferation and survival [14]. HeLa cells, derived from human cervical carcinoma, are widely used as an experimental model for evaluating the anticancer effects of natural compounds. In particular, lupeol has been reported to decrease HeLa cell proliferation and viability, induce S-phase cell-cycle arrest, and promote mitochondria-mediated apoptosis.

Among natural sources rich in triterpenes, the argan tree (*Argania spinosa* (L.) Skeels), a species belonging to the *Sapotaceae* family, holds considerable promise. Endemic to southwestern Morocco, the argan forest covers approximately 871,210 hectares, with an estimated density of 20 million trees and an annual fruit yield of about 500 kg per hectare [15]. The argan fruit is primarily valued for its kernels, which are used to produce edible argan oil [16,17]. Oil extraction generates several by-products, including approximately 43% pulp (pericarp), 52.6% hulls, and 2% argan press cake [18,19]. Several studies have examined the chemical composition of argan pulp, revealing a richness in phenolic compounds and carbohydrates such as cellulose, glucose, fructose, and sucrose [20,21] along with approximately 6% lipids. Notably, the unsaponifiable fraction of these lipids is particularly enriched in pentacyclic triterpenes, accounting for nearly 80% of this fraction. Identified compounds include erythrodiol, lupeol, α- and β-amyrin, taraxasterol, ψ-taraxasterol, betulinaldehyde, and betulin [22,23].

These triterpenes have been individually reported to exhibit potent biological activities, including anti-inflammatory, antioxidant, and anticancer effects. For instance, lupeol and amyrins have demonstrated the ability to suppress tumor growth and induce apoptosis across various cancer types, while erythrodiol and betulinaldehyde are known for their cytotoxic effects against tumor cells. The diversity and abundance of triterpenoids in argan pulp highlight its potential as a valuable and sustainable source of bioactive compounds for anticancer drug development. This chemical profile positions argan pulp not merely as an agricultural by-product, but as a promising raw material for targeted anticancer research.

The research aimed to explore the therapeutic potential of pentacyclic triterpene compounds isolated from argan pulp. GC/MS, ^1^H and ^13^C NMR and DEPT were employed for characterization. Selected in vitro and in silico assays were conducted to assess the cytotoxic effect against liver cancer (HepG2 cells), breast cancer (MCF-7 cells), and cervical cancer (HeLa cells).

## 2. Results and Discussion

Triterpenes are naturally occurring compounds with a wide range of biological activities. Their cytotoxic and chemopreventive properties make them promising candidates for alternative cancer treatments. The unsaponifiable fraction of argan pulp is particularly rich in triterpenes, notably amyrins, taraxastanes, lupeol, and erythrodiol. Five fractions were successfully recovered, with their respective proportions in the unsaponifiable matter as follows: F1 (carbohydrates), 14%; F2 (triterpenes), 48%; F3 (sterols), 2%; F4 (erythrodiol), 21%; and F5 (other compounds), 15% [24].

### 2.1. Characterizations

#### 2.1.1. GC/MS Analysis

**Figure 1:**
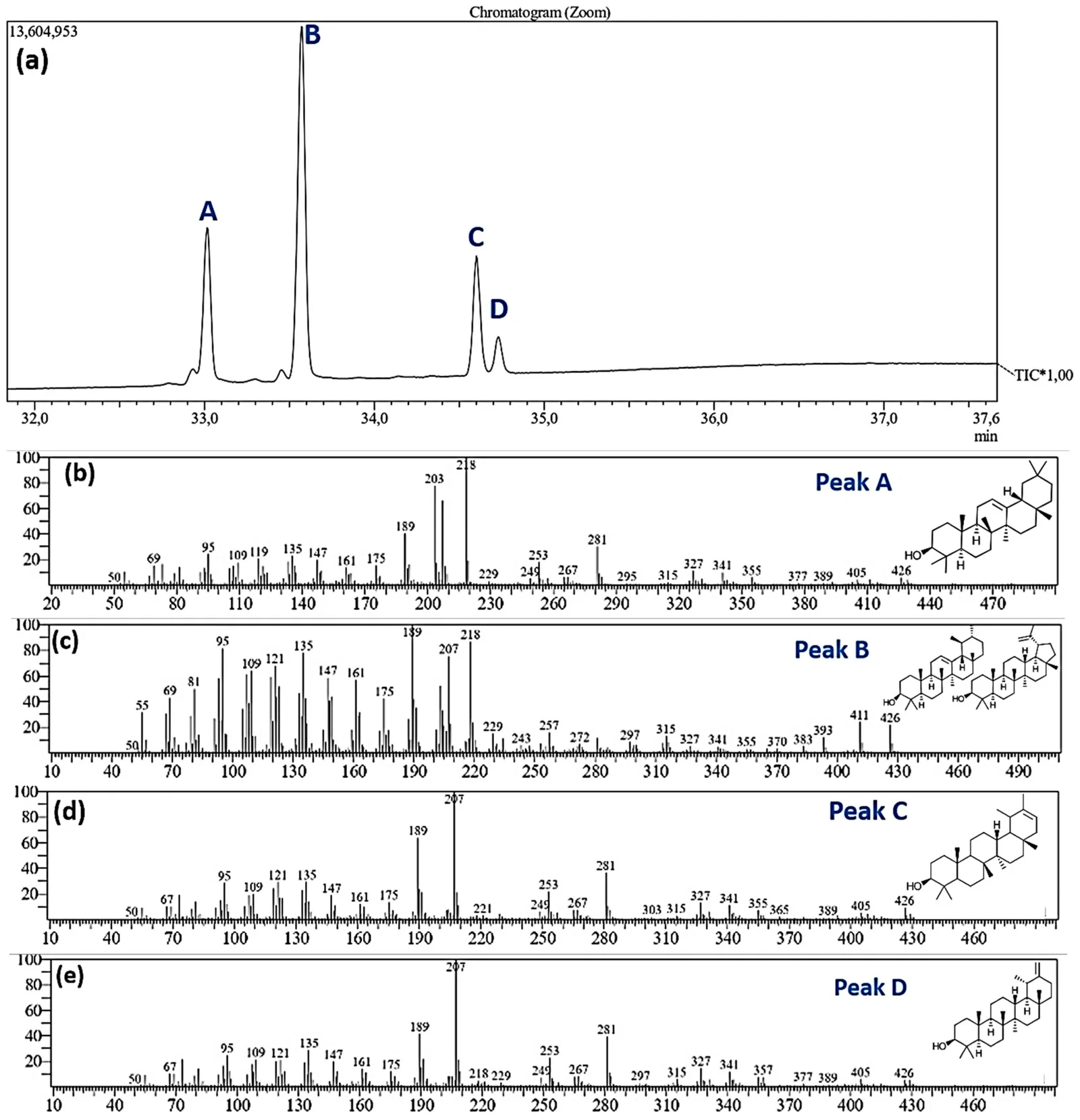
Gas chromatography–mass spectrometry characterization of the pentacyclic triterpenoid fraction (F2). (**a**) Enlarged total ion chromatogram showing the four main peaks, labeled A–D; (**b**) mass spectrum of peak A, assigned to β-amyrin; (**c**) mass spectrum of peak B, attributed to the co-elution of lupeol and α-amyrin; (**d**) mass spectrum of peak C, assigned to ψ-taraxasterol; (**e**) mass spectrum of peak D, assigned to taraxasterol. The proposed chemical structures of the corresponding triterpenoids are shown in the respective mass spectra.

The mixture of pentacyclic triterpenoids (Fraction 2) was obtained as a white powder. GC–MS analysis revealed four identifiable peaks, labeled A to D (Figure 1a). Peak A, representing 23.68% of the fraction F2 (Table S1), exhibited a retention time of 33.02 min. Its mass spectrum showed a molecular ion peak at *m*/*z* 426 (Figure 1b), corresponding to the molecular formula C_30_H_50_O. This formula indicates six degrees of unsaturation, five attributable to the pentacyclic carbon skeleton and one to a C=C double bond [25]. The mass spectrum also displayed fragment ions at *m*/*z* 218 (M − C_14_H_22_ − H_2_O) and *m*/*z* 203, resulting from a retro-Diels–Alder (RDA) cleavage involving the double bond between C-12 and C-13. According to Budzikiewicz (2015) and Lourenço et al. (2021) [26,27], this fragmentation pattern is characteristic of amyrins belonging to the oleanane and ursane series.

The RDA reaction of amyrins, generally followed by the loss of a methyl group at C-17, leads to the formation of an ion at *m*/*z* 203. A peak at *m*/*z* 189, which typically accompanies the ion at *m*/*z* 203, was also detected. Based on the observed fragmentation pattern, the ion at *m*/*z* 189 is attributed to double hydrogen transfer cleavage from the ion at *m*/*z* 218 (Figure S1). The overall mass spectral features indicate cleavage patterns characteristic of the oleanane and ursane families, suggesting that the compound is either β-amyrin or α-amyrin.

Distinguishing between β-amyrin (oleanane-type) and α-amyrin (ursane-type) using mass spectrometry alone is challenging, as their closely related cyclic backbones yield nearly identical mass spectra, differing mainly in the relative intensities of key fragment ions [28]. In the mass spectrum of β-amyrin, the ion at *m*/*z* 203 is typically about twice as intense as the ion at *m*/*z* 189, whereas in α-amyrin these two ions generally exhibit comparable intensities. As authentic standards of α- and β-amyrin were not available, identification was based on relative fragment intensities and reported GC retention times from the literature [29,30]. In the present analysis, the ion at *m*/*z* 203 was significantly more intense than that at *m*/*z* 189, supporting the identification of β-amyrin. Furthermore, GC data indicate that β-amyrin elutes earlier than α-amyrin [31,32,33]. Although α-amyrin may also be present in the mixture, it elutes after β-amyrin. Based on these observations, peak A was assigned to β-amyrin. Peak B, representing 66.77% of F2 (Table S1, Figure S2), exhibited a retention time of 33.56 min. The corresponding mass spectrum (Figure 1c) showed a molecular ion at *m*/*z* 426. Additional fragment ions at *m*/*z* 411 (M − CH_3_) and *m*/*z* 393 (M − CH_3_ − H_2_O) indicate the presence of a triterpene alcohol. The base peak at *m*/*z* 189 is characteristic of lupane, hopane, taraxastane, or euphane type triterpenes. The presence of diagnostic ions at *m*/*z* 383, 207, and 189 is consistent with lupeol (Figure S3) [34]. However, the simultaneous detection of ions at m/z 218 and 203, which are characteristic of amyrins, suggests that peak B likely represents a co-eluting mixture of lupeol and α-amyrin. The mass spectra of peaks C (Figure 1d) and D (Figure 1e) both exhibited a molecular ion at *m*/*z* 426, representing 3.88% and 0.72% of fraction F2, respectively (Table S1), corresponding to monohydroxylated triterpene alcohols with the molecular formula C_30_H_50_O. The dominant fragment ions at *m*/*z* 207 and 189 confirm the presence of ψ-taraxasterol (Figure S4) and taraxasterol (Figure S5) [35]. According to the literature, ψ-taraxasterol elutes earlier than taraxasterol under GC conditions [36,37]. Accordingly, peak C (Rt = 34.60 min) was assigned to ψ-taraxasterol, while peak D (Rt = 34.72 min) was attributed to taraxasterol.

**Figure 2:**
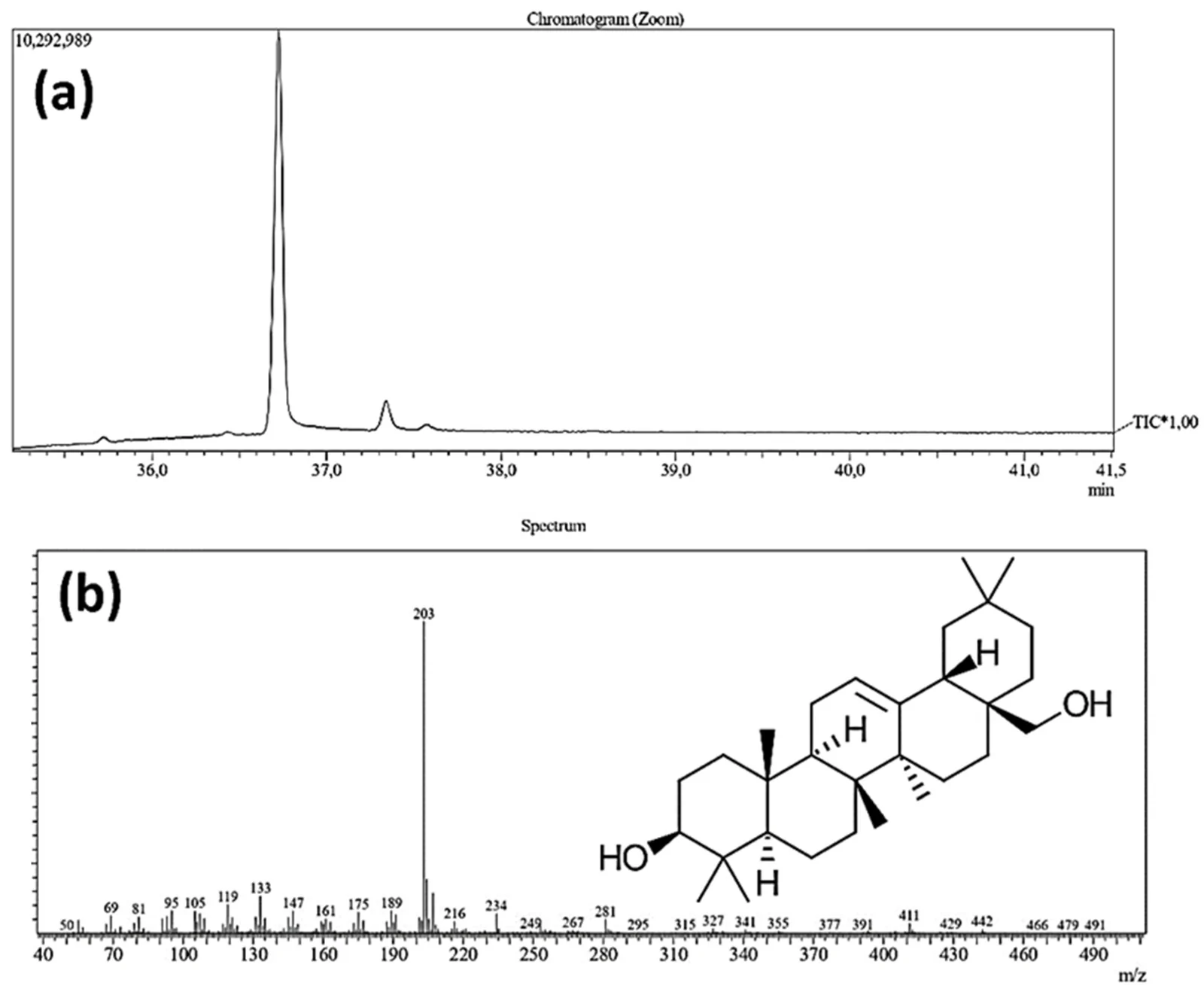
Gas chromatography–mass spectrometry characterization of fraction F4. (**a**) Enlarged total ion chromatogram showing the predominant peak at a retention time of 36.73 min; (**b**) mass spectrum of the main chromatographic peak, showing a molecular ion at *m*/*z* 442 and characteristic fragment ions consistent with erythrodiol.

Fraction F4 was obtained as an amorphous white powder. The chromatogram showed a dominant single peak at a retention time of 36.73 min (Figure 2a), accounting for approximately 90% of the total peak area (Figure S6, Table S2), indicating that F4 predominantly consists of a single compound with only trace-level impurities.

The electron impact (EI) mass spectrum of F4 (Figure 2b) displayed a molecular ion peak at *m*/*z* 442, consistent with the empirical formula C_30_H_50_O_2_. The fragmentation pattern revealed characteristic ions at *m*/*z* 442, 216, 203, 188, 133, 73, and 55, which are diagnostic for erythrodiol and are in good agreement with previously reported data [38,39,40]. A proposed fragmentation pathway for erythrodiol is presented in Figure S7. Taken together, the chromatographic purity and mass spectral features indicate that fraction F4 predominantly consists of erythrodiol. Although the structural assignments were supported by GC–MS and NMR data, further confirmation and accurate quantification would benefit from the use of authentic reference standards, which will be considered in future studies.

#### 2.1.2. NMR Spectra of Erythrodiol

^1^H-NMR (500 MHz, CHLOROFORM-D) δ (ppm) (Figure S8) showed peaks characteristic of a pentacyclic triterpenoid. A single olefinic proton signal (a triplet) was observed at δ_H_: 5.17 ppm and was assigned to H-12; it also revealed two resonant oxygenated protons at δ_H_: 3.55 ppm and δ_H_: 3.22 ppm. These were assigned to H-3 and H-28, respectively, indicating the presence of a hydroxyl moiety. Seven methyl peaks were also present at δ_H_ (ppm): 1.42, 1.15, 0.98, 0.91, 0.87, 0.85 and 0.77 for each 3H. ^13^C-NMR spectra of the compound revealed the presence of 30 carbon signals (Figure S9). ^13^C-NMR (125 MHz, CHLOROFORM-D) δ (ppm): 144.27 (C-13), 122.44 (C-12), 79.08 (C-3), 69.79 (C-28), 55.22 (C-5), 47.64 (C-9), 46.52 (C-19), 42.40 (C-18), 42.40 (C-14), 38.85 (C-1), 38.65 (C-4), 37.01 (C-17), 36.99 (C-10), 39.80 (C-8), 34.23 (C-21), 33.34 (C-29), 32.63 (C-7), 31.11 (C-22), 31.04 (C-20), 28.16 (C-23), 27.33 (C-2), 26.02 (C-27), 25.61 (C-15), 23.72 (C-30), 23.66 (C-11), 22.05 (C-16), 18.42 (C-6), 16.80 (C-26), 15.66 (C-24), 15.60 (C-25). The DEPT spectrum (Figure S10) shows 7 methyl carbons, 11 methylenes, 5 methines and 7 quaternary carbons. As a result, F4 was identified as erythrodiol (CAS No. 545-48-2). The isolation of erythrodiol as a pure compound in fraction 4 (F4), unlike the mixture of triterpenoids observed in fraction 2 (F2), can be attributed to differences in polarity and molecular structure. Erythrodiol is a dihydroxy (diol) triterpenoid with a molecular ion peak at *m*/*z* 442, while all compounds in F2 α-amyrin, β-amyrin, lupeol, taraxasterol, and ψ-taraxasterol are monohydroxylated triterpenes with identical molecular ion peaks at *m*/*z* 426 (C_30_H_50_O). The presence of an additional hydroxyl group in erythrodiol increases not only its mass but also its polarity, resulting in stronger interactions with the polar stationary phase during silica gel chromatography. As a result, erythrodiol was eluted later under the chromatographic conditions used, allowing for its efficient separation into the more polar fraction.

### 2.2. Cytotoxic Activity on HepG2, MCF-7, and HeLa Cell Lines

**Figure 3:**
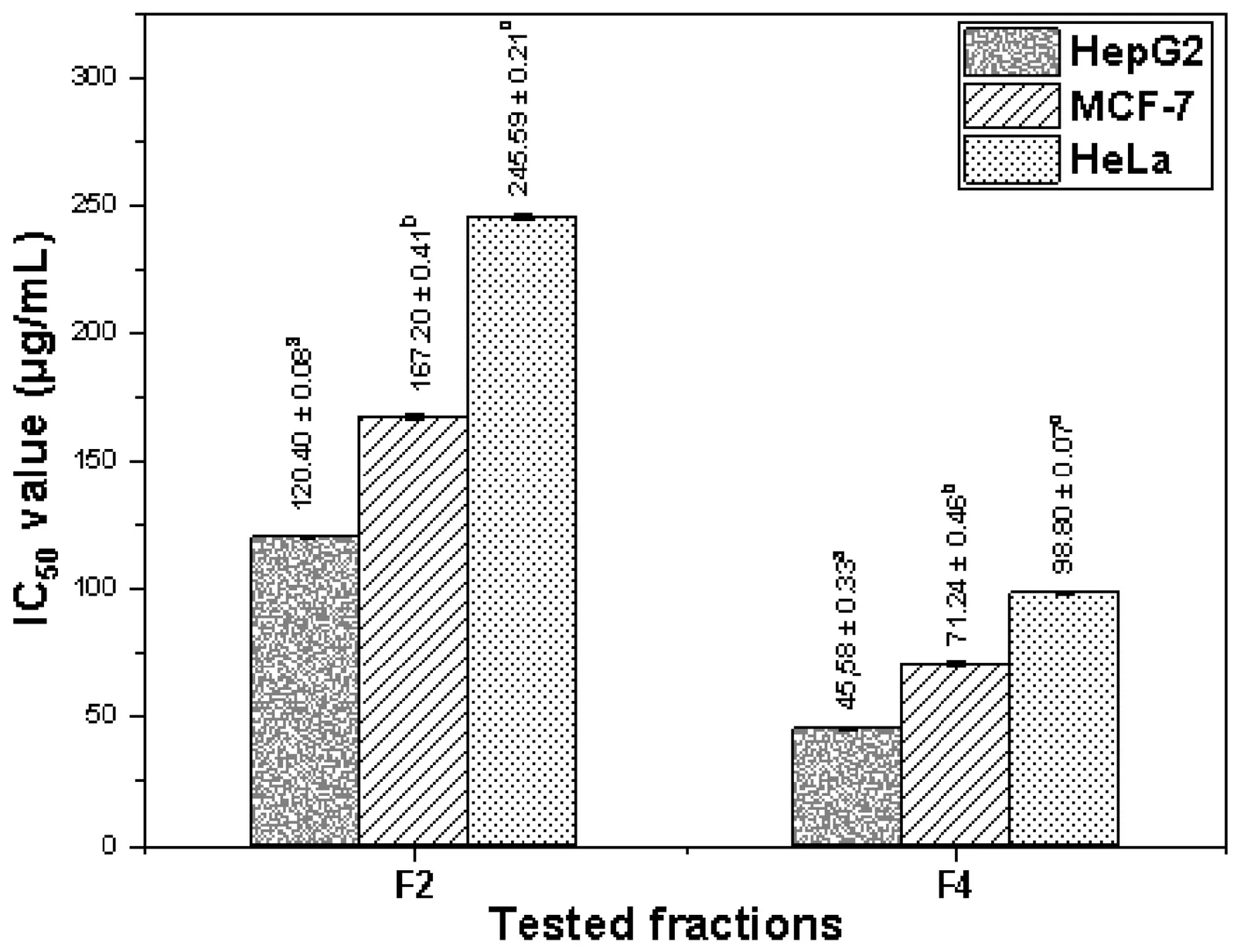
IC_50_ values of F2 and F4 fractions against HepG2, MCF-7, and HeLa cell lines. Values are expressed as mean ± standard deviation of three independent biological replicates (*n* = 3). Different lowercase letters within the same fraction indicate statistically significant differences among cell lines, as determined by one-way analysis of variance followed by Tukey’s honestly significant difference post hoc test (*p* < 0.05).

The cytotoxic effects of the different fractions on HepG2 (human hepatocarcinoma), MCF-7 (human breast cancer), and HeLa (human cervical cancer) cell lines were evaluated after 48 h of treatment. The concentrations resulting in 50% inhibition of cell growth (IC_50_) are presented in Figure 3.

Both F2 and F4 exhibited cytotoxic effects against the tested cancer cell lines, with significant differences in IC_50_ values (*p* < 0.05). The lowest IC_50_ value was observed in HepG2 cells, followed by MCF-7 and HeLa cells, suggesting a cell line-dependent response. Cells were treated for 24 h with the triterpenoids, as evidenced by significant differences in IC_50_ values (*p* < 0.05). This variability may be attributed to differences in cellular sensitivity as well as to the ability of triterpenes to modulate multiple signaling pathways involved in cell proliferation and survival [4]. Previous studies have reported that erythrodiol possesses antioxidant and anti-inflammatory properties, which contribute to apoptosis induction and inhibition of cell proliferation [41]. Erythrodiol exhibits dose-dependent antiproliferative and cytotoxic effects on the HepG2 cell line, with an IC_50_ value of 45.58 µg/mL, which is lower than the IC_50_ previously reported for erythrodiol-induced necrosis in HT-29 cells (48.8 µg/mL) [42]. However, other studies have reported even lower IC_50_ values (27.3 µg/mL) against HepG2 cells [43]. Furthermore, erythrodiol exhibits cytotoxic effects against MCF-7 and HeLa cell lines, with IC_50_ values of 71.24 µg/mL and 98.80 µg/mL, respectively. According to Allouche et al. (2011) [41], erythrodiol inhibits MCF-7 cell proliferation with IC_50_ values ranging from 25 to 50 µg/mL. However, its cytotoxic activity against human cervical adenocarcinoma (HeLa) cells has not yet been fully investigated. The F2 fraction, which is rich in β-amyrin, α-amyrin, and lupeol, exhibited moderate cytotoxic activity, with IC_50_ values of 122.6, 167.8, and 212.7 µg/mL against HepG2, MCF-7, and HeLa cell lines, respectively. These findings are consistent with those reported by Khunoana et al. [44] who demonstrated that a fraction containing β-amyrin and lupeol isolated from *Ptaeroxylon obliquum* leaf extracts exhibited similar IC_50_ values against the same cell lines. In contrast, β-amyrin and lupeol tested as pure compounds showed higher cytotoxicity against MCF-7 cells, with IC_50_ values of 15.5 µg/mL and 17 µg/mL, respectively [45]. However, Teclegeorgish et al. (2020), reported that lupeol exhibited no cytotoxicity against MCF-7 cells (IC_50_ > 500 µM) [46]. Similarly, Lima et al. (2014) [47] reported that two fractions containing α-amyrin and β-amyrin exhibited weak cytotoxic effects, with IC_50_ values of 34.8 and 38.2 µg/mL, respectively. However, when tested individually, α-amyrin and β-amyrin demonstrated potent cytotoxic activity against MCF-7 cells, with IC_50_ values of 2.35 and 2.48 µg/mL, respectively.

In the present study, cytotoxic effects were evaluated only in cancer cell lines, and the effects on normal (non-cancerous) cells were not investigated. Therefore, the selectivity of the tested fractions toward cancer cells cannot be established. Further studies are required to assess their cytotoxicity in normal cell models (HEK293, HUVEC, or primary cells) and to determine their selectivity index. However, further in vivo studies are required to validate its effects on tumor growth and to elucidate its interactions with the tumor microenvironment, particularly in liver and breast cancers. Moreover, the differential responses observed among HepG2, MCF-7, and HeLa cells suggest opportunities for the development of targeted treatment strategies. Erythrodiol may also be optimized through combination therapies that target complementary signaling pathways [48,49]. To more accurately assess the cytotoxic potential of the F2 fraction, additional studies are needed to isolate its individual constituents and evaluate their cytotoxic effects independently. Overall, cytotoxicity exhibited by triterpenes underscores their promise as natural compounds for the development of novel cancer therapeutics.

The evaluation of the cytotoxic activity of triterpenes isolated from argan pulp revealed different activity profiles between the two fractions tested. Fraction F4, mainly composed of erythrodiol, exhibited significant and dose-dependent antiproliferative effects against HepG2, MCF-7, and HeLa cell lines, whereas fraction F2, enriched in α/β-amyrin and lupeol, showed more moderate cytotoxic activity. When considered alongside previously reported data (Table 1), erythrodiol extracted from *Olea europaea* exhibited comparable activity, with a 50% inhibitory concentration (IC_50_) of 48.8 µg/mL against HT-29 colorectal adenocarcinoma cells [50]. Similarly, Peñas-Fuentes et al. [43] reported an IC_50_ value of 15.3 µg/mL in HepG2 cells using commercial erythrodiol. These findings support the consistency of erythrodiol’s antiproliferative effects across different experimental systems and cancer cell types. In contrast, β-amyrin, a constituent of fraction F2, has been reported to exhibit variable cytotoxic activity depending on the cell line and experimental conditions. For instance, [51] demonstrated strong antiproliferative effects of β-amyrin in HepG2 cells (IC_50_ ≈25 µg/mL), whereas [52] reported considerably lower activity in the same cell line (IC_50_ = 206 µg/mL), along with moderate cytotoxicity toward noncancerous HEK293 cells (IC_50_ = 156 µg/mL). Similarly, α/β-amyrin mixtures isolated from *Mesua ferrea* exhibited stronger cytotoxic effects against MCF-7 cells (IC_50_ = 28.45 µg/mL) [53] than those observed for the F2 fraction in the present study, suggesting that synergistic interactions or structural variations in side chains may influence cytotoxic potency. These discrepancies highlight the strong influence of compound purity, experimental conditions, and cell type on the observed cytotoxic activity of triterpenes. Lupeol, another component of fraction F2, is generally considered to exhibit low cytotoxicity. Previous studies have shown that lupeol isolated from *Grewia lasiocarpa* displayed minimal antiproliferative activity, with IC_50_ values exceeding 1000 µg/mL against HeLa and MCF-7 cells [54], indicating a limited contribution to the overall cytotoxicity of the F2 fraction. However, some studies have reported enhanced cytotoxic activity for chemically modified lupeol derivatives, suggesting that structural modifications may significantly improve its biological efficacy. Overall, as summarized in Table 1, the cytotoxic activity of pentacyclic triterpenes varies widely depending on the compound, its purity, the tested cell line, and experimental conditions. Taken together, these findings suggest that erythrodiol may exert the most pronounced cytotoxic activity among the triterpenes isolated from *Argania spinosa*. The comparatively lower activity of the F2 mixture may be attributed to the dilution of active constituents and potential synergistic or antagonistic interactions among its components, although this remains to be further investigated. The observed dose-dependent effects and induction of apoptosis further highlight the biological activity of the triterpenes identified in this study. However, additional investigations are required to further explore their potential, including the evaluation of other biological activities and their effects on a broader range of cancer cell lines. Importantly, the utilization of argan pulp, an abundant by-product of argan oil production, offers notable ecological and economic advantages.

### 2.3. Flow Cytometry (Annexin V–FITC/PI)

**Figure 4:**
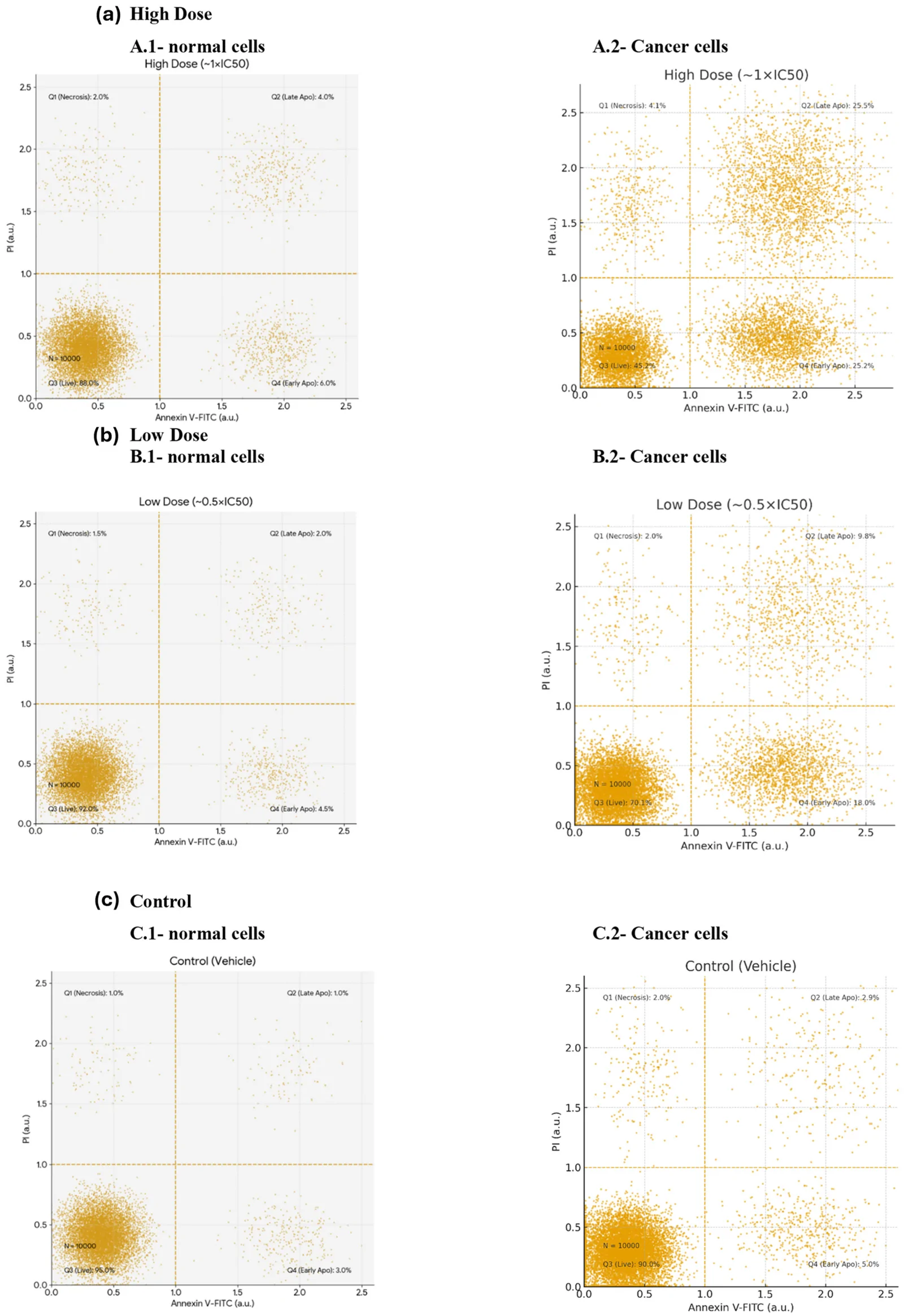
Representative Annexin V–FITC/PI flow-cytometry plots of THLE-2 (non-tumor cells) and HepG2 (cancer cells) following treatment with fraction F4. Panel (**a**) shows cells treated with the high dose of F4 (45.58 µg/mL, ~1× IC50), with subpanel (**A.1**) representing THLE-2 cells and (**A.2**) representing HepG2 cells. Panel (**b**) shows cells treated with the low dose of F4 (22.79 µg/mL, ~0.5× IC50), with (**B.1**) THLE-2 and (**B.2**) HepG2. Panel (**c**) shows vehicle-treated control cells (0 µg/mL), with (**C.1**) THLE-2 and (**C.2**) HepG2. The numbers in each quadrant indicate the percentage of necrotic (Q1), late apoptotic (Q2), viable (Q3), and early apoptotic (Q4) cells.

The Annexin V–FITC/PI assay showed that the non-tumor THLE-2 cells maintained high viability after treatment with F4 (Figure 4). Cell viability decreased slightly, from 95.0% in the control group (vehicle) to 92.0% and 88.0% after treatment with 22.79 and 45.58 µg/mL of F4, respectively. Most cells therefore remained in the viable cell quadrant (Annexin^−^/PI^−^, Q3). Total apoptosis (Q2 + Q4) increased slightly, from 4.0% in the control group (solvent) to 6.5% and 10.0% at concentrations of 22.79 and 45.58 µg/mL, respectively. Necrotic cells (Q1) remained few in number, accounting for 1.0%, 1.5%, and 2.0% under the corresponding conditions.

Under the same exposure conditions, HepG2 cells exhibited a marked, dose-dependent increase in apoptosis accompanied by a progressive decrease in viable cells. Using fraction F4 as the representative fraction illustrated in Figure 4, control (vehicle-treated) cells exhibited high viability (Q3 = 90.0%) with minimal apoptosis (total apoptosis, Q2 + Q4 = 7.9%). Fraction F2 showed the same overall directionality in the apoptotic response, although with a lower magnitude under the tested conditions. Treatment with F4 at the low dose (22.79 µg/mL; 0.5× IC_50_) reduced cell viability to 70.09% and increased total apoptosis to 27.89%, predominantly driven by early apoptotic cells (Q4 = 18.04%) with a moderate contribution from late apoptosis (Q2 = 9.85%). At the high dose (45.58 µg/mL; 1× IC_50_), cell viability further declined to 45.18%, while total apoptosis reached 50.68%, with early and late apoptotic populations being similarly represented (Q4 = 25.19% and Q2 = 25.49%), indicating progression toward late-stage apoptosis at higher concentrations. Quadrant analysis confirmed a gradual shift from viable cells (Q3) toward early apoptosis (Q4) and subsequently late apoptosis (Q2) with increasing dose. In contrast, necrotic cells (Q1) remained consistently low across all conditions (2.04% in control, 2.02% at the low dose, and 4.14% at the high dose). This pattern indicates that cell death occurred predominantly through apoptotic rather than necrotic mechanisms. Overall, control samples showed high cell viability with minimal apoptotic staining, low-dose treatment was characterized by a predominance of early apoptotic cells, and high-dose treatment resulted in a pronounced loss of viable cells with a balanced distribution of early and late apoptosis. These cytometric findings demonstrate a predominantly apoptotic phenotype; however, Annexin V–FITC/PI analysis alone does not establish the underlying molecular mechanisms of apoptosis. Targeted validation experiments, including mitochondrial membrane potential analysis (JC-1 or TMRE), intracellular ROS detection (DCFH-DA), PARP and caspase cleavage assays, and evaluation in additional non-tumor cell models, would further strengthen the mechanistic interpretation. Taken together, the flow cytometry data illustrate a clear dose–response relationship, with fraction F4 (erythrodiol) inducing the strongest apoptotic response, while fraction F2 (mixed triterpenes) shows a similar trend with a comparatively lower increase in total apoptosis.

### 2.4. In Silico Prediction of Physicochemical and Pharmacokinetic Profiles

The predicted physicochemical properties indicated that all four compounds complied with Lipinski’s rule of five (Table 2). Their molecular weights were below 500 g/mol, molar refractivity values were below 140 Å^3^, LogP values were lower than 5, and the numbers of hydrogen-bond acceptors and donors were below 10 and 5, respectively [57,58,59]. These results suggest physicochemical properties compatible with favorable drug-like behavior.

The predicted absorption, distribution, metabolism, excretion, and toxicity properties are summarized in Table 3.

All compounds showed high predicted human intestinal absorption, with values exceeding 95%. The predicted blood–brain barrier and central nervous system permeability parameters suggested a potential ability to penetrate biological barriers, although differences were observed among the compounds. None of the compounds was predicted to inhibit the major cytochrome P450 isoforms investigated, namely CYP1A2, CYP2C9, CYP2C19, CYP2D6, and CYP3A4. Moreover, all compounds were predicted to be negative for Ames mutagenicity and skin sensitization. However, compound C4 was predicted to exhibit potential hepatotoxicity, indicating that this compound may require further toxicological evaluation.

**Figure 5:**
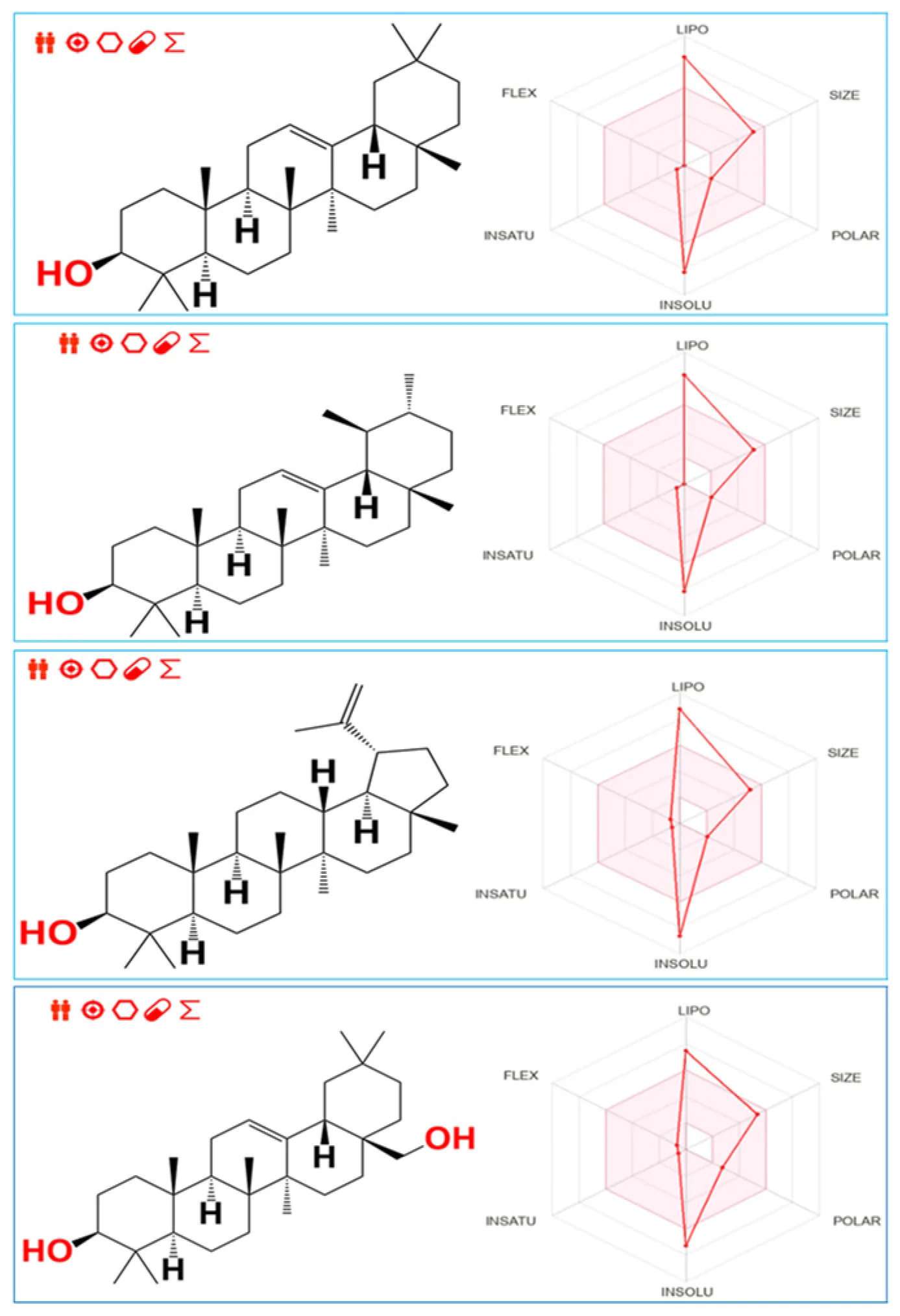
Bioavailability radars of α-amyrin, β-amyrin, lupeol, and erythrodiol, respectively.

The bioavailability radar plots further indicated generally favorable oral drug-like properties, as the physicochemical profiles of the compounds largely fell within the optimal bioavailability region for small molecules [60,61] (Figure 5).

### 2.5. Molecular Docking Analysis

**Figure 6:**
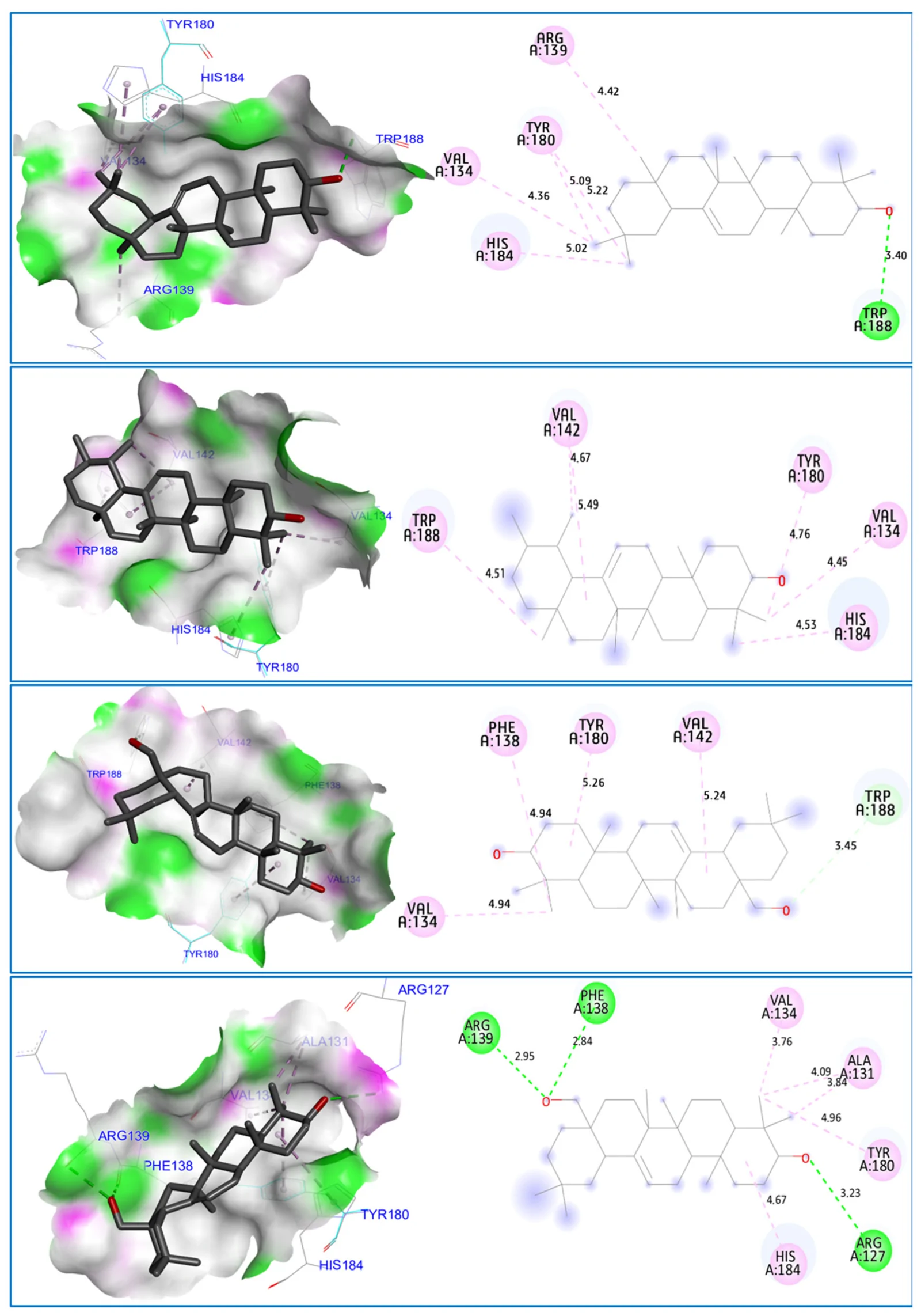
Produced interactions in 2D and 3D views for α-amyrin, β-amyrin, lupeol, and erythrodiol in complex with 6O0K protein.

Molecular docking analyses were performed to investigate the potential interactions of the four triterpenes with three cancer-related molecular targets: BCL-2 co-crystallized with venetoclax (PDB ID: 6O0K), the human estrogen receptor alpha ligand-binding domain (PDB ID: 3ERT), and the HPV16 E6 oncoprotein in complex with the IRF3 LxxLL motif (PDB ID: 6SJA). The candidate compounds were docked to the active sites of 6O0K encoded protein, responsible for HepG2 cells [62], revealing common intermolecular interactions including three alkyl bonds detected with Tyr180, Val134, and His184 amino acid residues, and the more common hydrogen bond fixed towards Trp188 amino acid residue, in addition to three hydrogen bonds produced for the erythrodiol compound in complex with Arg127, Phe138, and Arg139 amino acid residues, as presented in Figure 6.

**Figure 7:**
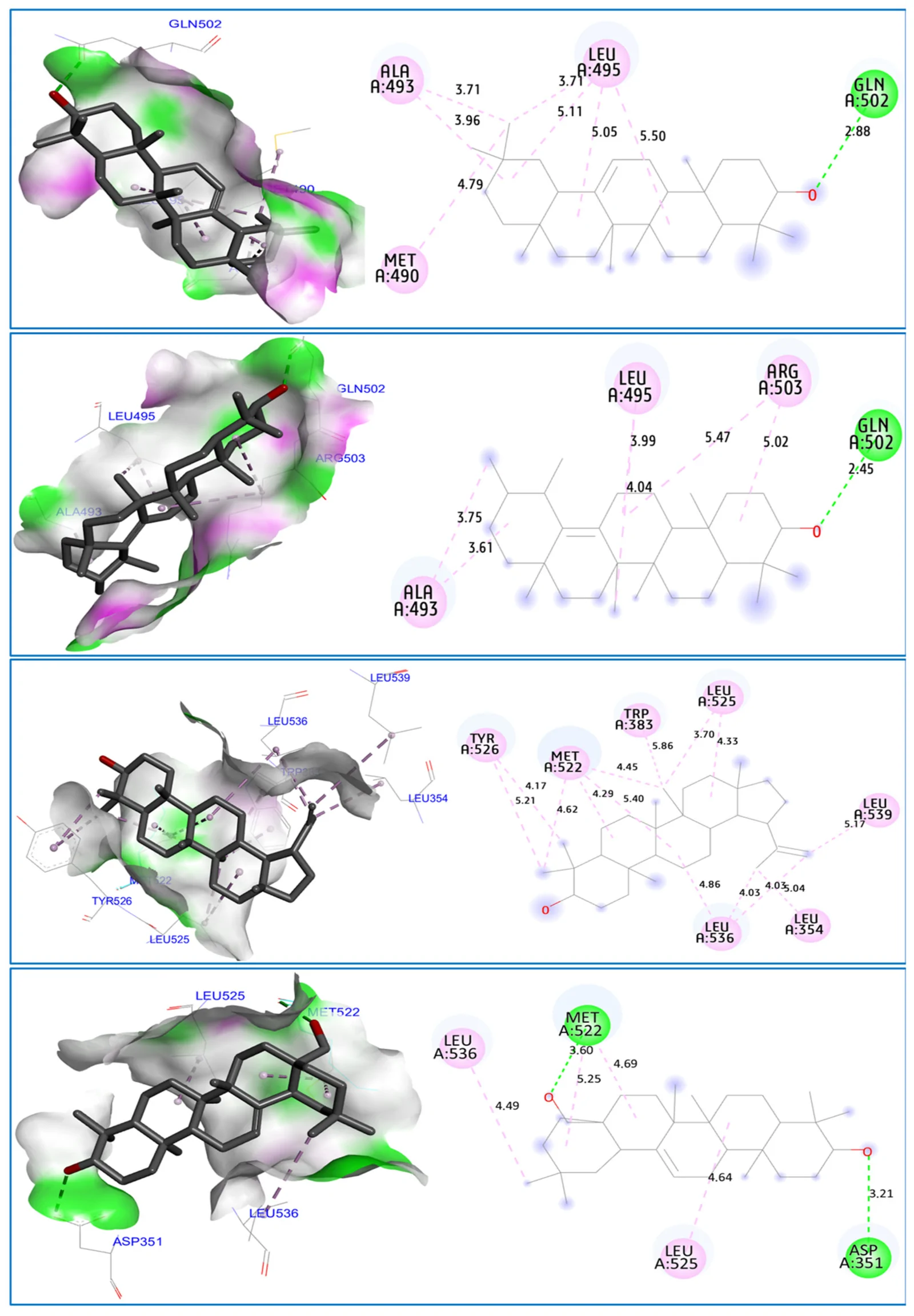
Produced interactions in 2D and 3D views for α-amyrin and β-amyrin, lupeol, and erythrodiol in complex with 3ERT protein.

The same examined compounds were secondly docked to 3ERT.pdb protein targeting MCF-7 cells [63], revealing two common hydrogen bonds detected by α- and β-amyrins, towards Gln502 active site in A chain, and more than two hydrogen bonds produced by erythrodiol molecule in complex with Met522 and Asp351 amino acid residues, as shown in Figure 7. These interactions suggest that the triterpenes can establish favorable contacts within the receptor-binding pocket.

**Figure 8:**
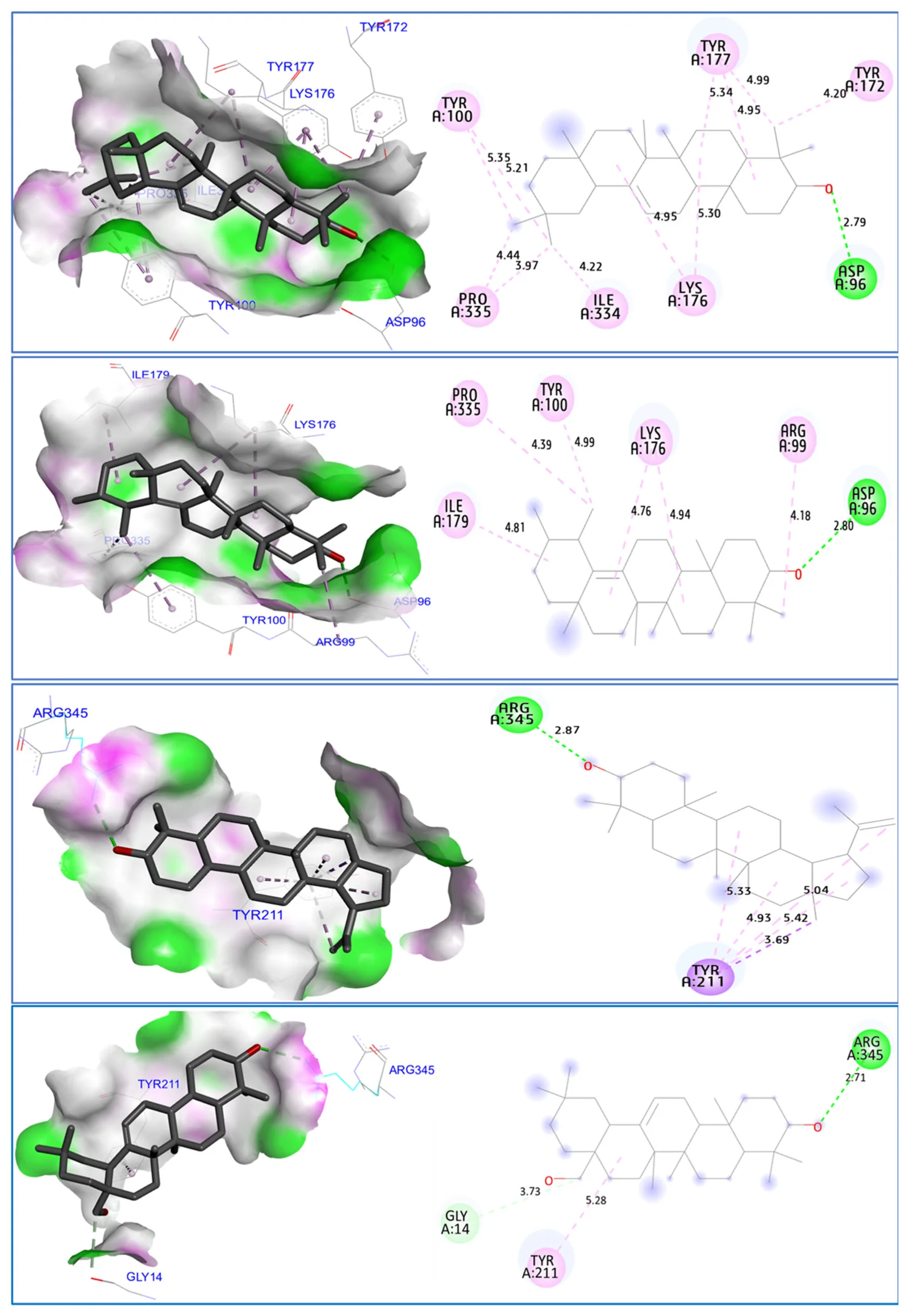
Produced interactions in 2D and 3D views for α-amyrin, β-amyrin, lupeol, and erythrodiol in complex with 6SJA protein.

Finally, the four triterpenes were docked against the HPV16 E6 oncoprotein [64]. Both α-amyrin and β-amyrin formed a hydrogen bond with Asp96, together with alkyl interactions involving Tyr100, Lys176, and Pro335. Lupeol and erythrodiol showed a common hydrogen-bond interaction with Arg345, while Tyr211 was also involved in interactions with the docked ligands (Figure 8). Overall, the docking results indicate that the investigated triterpenes can establish multiple favorable interactions with the selected cancer-related protein targets.

### 2.6. Docking Validation Protocol

To validate the molecular docking protocol, the native co-crystallized ligands were re-docked into their respective protein-binding sites [65]. The docking grids were centered on the crystallographic binding sites and defined to include the key amino acid residues involved in ligand recognition. The same scoring function, search algorithm, and docking parameters used for the investigated triterpenes were applied during the validation procedure.

Multiple conformations were generated for each ligand, and the optimal docking pose was selected based on binding affinity and agreement with the experimentally observed binding orientation. The reliability of the docking protocol was assessed by calculating the root-mean-square deviation between the heavy atoms of the re-docked and crystallographic ligand conformations. Values below 2.0 Å were considered indicative of successful reproduction of the experimental binding mode The resulting root-mean-square deviation values were 0.27 Å for venetoclax in BCL-2 (6O0K), 1.25 Å for 4-hydroxytamoxifen in the estrogen receptor alpha (3ERT), and 1.86 Å for the IRF3 LxxLL motif in the HPV16 E6 complex (6SJA). All values were below the predefined threshold of 2.0 Å, confirming the suitability of the docking protocol for the investigated targets (Figure S11). In addition, the calculated binding energies of the investigated triterpenes were comparable to, and in several cases more favorable than, those obtained for the corresponding co-crystallized ligands (Table 4).

## 3. Materials and Methods

### 3.1. Chemical Reagents and Measurements of Spectral Data

All chemical reagents were of analytical grade and supplied by Sigma-Aldrich, St. Louis, MO, USA. The pure product of erythrodiol was analyzed using Chloroform-D as the solvent on a Bruker Avance III NMR apparatus (Bruker Corporation, Billerica, MA, USA), which operates at a 1H resolution of 500 MHz and a 13C resolution of 125 MHz in this NMR analysis. 1H NMR spectra were recorded with 32 scans and a relaxation delay of 5 s. 13C NMR spectra were recorded with 2048 scans and a relaxation delay of 2 s. DEPT-135 13C NMR spectra were acquired with 1024 scans and a relaxation delay of 2 s. Chemical shifts are reported in (δ) units and expressed in parts per million (ppm), with the reference standard being tetramethylsilane (TMS). GC-MS analysis was conducted using a TRACE GC-ULTRA system coupled with a Polaris Q quadrupole mass detector (Thermo-Fisher Scientific, Waltham, MA, USA), equipped with an HP-5MS capillary column (50 m × 0.32 mm i.d., 1.25 µm film thickness) (Agilent Technologies, Santa Clara, CA, USA). The oven temperature was programmed from 40 °C to 280 °C at 5 °C/min. Helium served as the carrier gas at 1 mL/min. Samples (1 μL) were injected in split mode (1:40), with injector and detector temperatures set at 250 °C and 200 °C, respectively. Ionization was performed in EI mode at 70 eV. Compounds were identified by comparing retention times and mass spectra with the NIST-MS Library (Version 2.0).

### 3.2. Sample Preparation

Argan pulp (1 kg) was supplied by an argan oil-producing cooperative located in the Taroudant region of south-west Morocco. The samples were collected in (October 2024) from five batches from Taroudant region air-dried at room temperature for two weeks. The dried pulp was ground using a standard kitchen grinder, then sieved through a 1 mm mesh sieve to obtain a uniform particle size, and then stored at 4 °C until extraction. Eighty grams was then extracted with 240 mL petroleum ether in a Soxhlet apparatus at 60 °C for 6 h, followed by filtration on Whatman filter paper (number 1). A rotary evaporator concentrated the extract to give a brownish-yellow lipid extract. The lipid yield was 6.5%. Approximately 2.5 g of lipids were accurately weighed into a 250 mL round-bottomed flask; then 100 mL of 1 M potassium hydroxide ethanolic solution (prepared by dissolving 5.6 g potassium hydroxide in 100 mL 95% ethyl alcohol) was added. The mixture was then heated under reflux for one hour, and hot distilled water was added to stop the reaction. The unsaponifiable matter was then extracted with petroleum ether (4–5 times until complete exhaustion of the organic phase). The resulting organic phase, containing the unsaponifiable matter, was washed several times with a solution of water and ethanol (90/10). After evaporation of the solvent, the remaining yellow powder, representing the unsaponifiable matter, was weighed, giving an unsaponifiable matter yield of 23%.

### 3.3. Separation Procedure

The unsaponifiable matter (3 g) was dissolved in ethyl acetate/methanol (80/20) and approximately 10 g of silica gel (230–400 mesh, 40–63 µm; Cat. No. 96671) was added for pre-adsorption. The solvent was removed using a rotary evaporator and the dry mixture was transferred to a silica gel chromatography column (approximately 50 cm in length and 2.5 cm in inner diameter) containing 50 g of silica that had been soaked overnight in 100 mL of hexane. The column was first eluted with 100 mL of 100% petroleum ether, followed by a gradient of petroleum ether and ethyl acetate in the ratios 95:5, 90:10, 85:15, 80:20, 75:25, and 70:30 (100 mL each). Finally, the column was washed with 100 mL of pure ethyl acetate to ensure complete elution of compounds. Five fractions were collected in 15 mL centrifuge tubes and analyzed by thin-layer chromatography (TLC) on silica gel 60 F254 plates (Merck, Darmstadt, Germany). TLC profiling was performed using petroleum ether: ethyl acetate (80:20 and 75:25) as the mobile phases. Spots were visualized under a UV lamp at 254 and 366 nm, after spraying the plates with a 0.05% fluorescein solution in ethanol as a detection reagent. Fractions with similar TLC profiles were pooled, and the solvents were evaporated to recover the separated compounds, which were then stored at 4 °C until further analysis.

### 3.4. Cytotoxicity In Vitro

The HepG2 and HeLa were obtained from the American Type Culture Collection (ATCC, Manassas, VA, USA), and MCF-7 was purchased from Applied Biological Materials Inc. (abm, Richmond, BC, Canada). MCF-7, HeLa and HepG2 cell lines were maintained with RPMI 1640 medium supplemented with 5% heat-inactivated fetal bovine serum, 1% penicillin G-streptomycin, and 0.2% of l-glutamine. Incubation was performed at 37 °C in humidified atmosphere containing 5% CO_2_.

In this assay, cell viability was evaluated using the MTT assay. In brief, cells were washed with phosphate-buffered saline (PBS) and seeded into 96-well microplates. MCF-7 and HeLa cells were seeded at a density of 1 × 10^4^ cells/well, whereas HepG2 cells were seeded at 2 × 10^4^ cells/well, followed by overnight incubation. Subsequently, 100 μL of culture medium containing serial concentrations of each sample (0, 25, 50, 75, 100, 150, 200, 250, and 400 μg/mL) was added. DMSO was used as the vehicle, and its final concentration did not exceed 0.2% (*v*/*v*) in any treatment. A DMSO vehicle control was included. After 48 h of incubation, 20 μL of MTT solution (5 mg/mL) was added to each well and incubated under the same conditions for an additional 4 h. The supernatant (150 μL) was carefully removed and replaced with 150 μL of acidified isopropanol (0.04 N HCl in isopropanol) to solubilize the formazan crystals. Absorbance was then measured at 540 nm using a Multiskan^®^ microplate reader (Thermo Fisher Scientific, Waltham, MA, USA). Cell viability was expressed as a percentage relative to the control using the formula: Viability (%) = 100 × (Ai/A0), where A0 represents the absorbance of control cells and Ai represents the absorbance of treated cells. The half-maximal inhibitory concentration (IC_50_) was determined by nonlinear regression analysis and corresponded to the concentration producing 50% inhibition of cell viability.

### 3.5. Flow Cytometry (Annexin V–FITC/PI)

Apoptosis in HepG2 cancer cells and THLE-2 non-tumor cells was evaluated by flow cytometry using dual Annexin V–FITC/propidium iodide (PI) staining. Cells were treated for 24 h with fraction F4 (erythrodiol) at 22.79 and 45.58 µg/mL, with DMSO (≤0.5% *v*/*v*) used as the vehicle control. For HepG2 cells, these concentrations corresponded to 0.5× IC_50_ and 1× IC_50_, respectively, based on the F4 IC_50_ value of 45.58 µg/mL. For THLE-2 cells, no IC_50_ value was determined; therefore, the same concentrations (22.79 and 45.58 µg/mL) were applied as absolute concentrations and were not expressed as 0.5× IC_50_ or 1× IC_50_. These concentrations were selected to allow direct comparison between HepG2 and THLE-2 cells. Both adherent and floating cells were collected, washed with cold PBS, and resuspended in Ca^2+^-containing binding buffer before incubation with Annexin V–FITC and PI for 15 min at room temperature in the dark, according to the manufacturer’s instructions. Data were acquired within 1 h using a 488 nm flow cytometer (FITC: ~530/30 nm; PI: >600 nm). Compensation was performed using single-stained controls, and debris and doublets were excluded using FSC/SSC and FSC-A/FSC-H gating. At least 10,000 singlet events were collected per sample. Quadrants were defined as Q3 (Annexin V^−^/PI^−^, viable cells), Q4 (Annexin V^+^/PI^−^, early apoptotic cells), Q2 (Annexin V^+^/PI^+^, late apoptotic cells), and Q1 (Annexin V^−^/PI^+^, necrotic cells). Total apoptosis was calculated as the sum of early and late apoptotic populations (Q2 + Q4). Staurosporine (0.5–1 µg/mL for 4–6 h) was included as a positive control when required. Data were analyzed using FlowJo (Version 10.10.1) and are presented as mean ± SD from three independent biological experiments. Statistical comparisons were performed using one-way ANOVA followed by Tukey’s post hoc test, with statistical significance set at α = 0.05.

### 3.6. In Silico Study

In this study, an in silico analysis was conducted to predict the physicochemical and pharmacokinetic properties associated with absorption, distribution, metabolism, excretion, and toxicity (ADMET) using the SwissADME and pkCSM online platforms [66,67]. Additionally, a molecular docking study was performed on three selected target proteins using AutoDockTools-1.5.6 [68,69,70]. For docking, the four chemical compounds were prepared and docked to each protein using chain A, which contained the co-crystallized native ligand, after being converted from PDB to PDBQT format. The co-crystallized ligands and crystallographic water molecules were removed, and the Gasteiger charges were added, while non-essential heteroatoms were excluded during receptor preparation. Polar hydrogen atoms were equally added, and appropriate protonation states and configurations of ligands were retained. Initial ligand structures were optimized for geometry before docking, and grid parameters for each target are listed in Table 5. A genetic algorithm was employed for docking, with 10 GA runs, a population of 150, a maximum of 2,500,000 energy evaluations, and a maximum of 27,000 generations. Mutation and crossover rates were 0.02 and 0.8, respectively, and a two-point crossover mode was adopted. Redocking validation was run on AutoDockTools-1.5.7rc1. Finally, BIOVIA Discovery Studio Visualizer 2021 was used to visualize the protein–ligand interactions in two and three dimensions [68,69,70].

## 4. Conclusions

This study focuses on the cytotoxicity of pentacyclic triterpenes present in the unsaponifiable lipid fraction extracted from argan pulp. The unsaponifiable fraction, separated by open-column chromatography, revealed two triterpene fractions: the first, F2, comprising α-amyrin, β-amyrin, lupeol, ψ-taraxasterol and taraxasterol, and the second, F4, containing erythrodiol. Both fractions showed cytotoxic activity on all tested cell lines, with the greatest sensitivity observed in HepG2 cells, followed by MCF-7 and HeLa cells. In parallel, Annexin V–FITC/PI flow cytometry in HepG2 and THLE-2 cells indicated that F4 treatment was associated with a predominantly apoptotic response in HepG2 cells, with necrosis remaining marginal. Among the compounds tested, erythrodiol demonstrated the greatest activity, and molecular docking suggested favorable interactions with key residues of BCL-2 family proteins, the human estrogen receptor-α, and the HPV16 E6 oncoprotein, providing preliminary mechanistic insights consistent with an apoptotic response. However, the predicted binding energies represent computational estimates and should not be interpreted as actual in vivo binding affinities. Further molecular dynamics simulations and experimental validation are required to confirm the stability and biological relevance of these ligand–protein interactions. Although these results provide preliminary insights into the cytotoxic potential of triterpene-rich fractions, further research is needed to confirm their biological activity. This includes evaluating the individual cytotoxic activities of the identified triterpenes, as well as conducting a more comprehensive assessment of their selectivity toward cancer cells versus healthy cells, particularly using models of healthy human liver.

Looking ahead, in vivo validation should prioritize pharmacokinetics, biodistribution, and therapeutic efficacy of erythrodiol and related triterpenes to translate these in vitro findings into clinically relevant applications.

## Figures and Tables

**Table 1:** Studies on the cytotoxicity of α- and β-amyrins, lupeol, and erythrodiol.

Article	Source/Study	Compounds	Cell Line(s) Tested	Cytotoxic Activity/Cell Line IC_50_ (μg/mL)	Reference
This study	Argan pulp (*Argania spinosa* (L.) Skeels)	F4 (erythrodiol)	HepG2	45.58	Text
			MCF-7	71.24	
			HeLa	98.80	
		F2 (mixture: α/β-amyrin, lupeol, ψ-taraxasterol, taraxasterol)	HepG2	122.6	
			MCF-7	167.8	
			HeLa	212.7	
Phytochemistry, cytotoxicity and apoptosis studies of β-sitosterol-3-o-glucoside and β-amyrin from *Prunus africana*	*Prunus africana* (leaves & bark)	β-Amyrin	HepG2	206	[52]
			Caco-2 (cancer)	81	
			HEK293 (non-cancer)	156	
Erythrodiol, a natural triterpenoid from olives, has antiproliferative and apoptotic activity in HT-29 human adenocarcinoma cells	From olives (*Olea europaea*)	Erythrodiol	HT-29 human colorectal adenocarcinoma cells	48.8	[50]
The Pharmaceutical Potential of α- and β-Amyrins.	*Mesua ferrea* stem bark	α, β-Amyrin mixture	MCF-7 breast cancer	28.45	[53]
Effects of Erythrodiol on the Antioxidant Response and Proteome of HepG2 Cells		Erythrodiol	HepG2 (liver cancer) cells	15.3	[43]
Isolation of lupeol from *Grewia lasiocarpa* stem bark: Antibacterial, antioxidant, and cytotoxicity activities	*Grewia lasiocarpa* stem bark	Lupeol	HEK293	ND	[54]
			HeLa	>1000	
			MCF-7	>1000	
Antitumor effects of beta-amyrin in Hep-G2 liver carcinoma cells are mediated via apoptosis induction, cell cycle disruption and activation of JNK and P38 signalling pathways	_	β-Amyrin	HepG2	25	[51]
			non-cancer FR-2 cells.	75	
Cytotoxic Activity of Some Lupeol Derivatives	_	Lupeol	HeLa	37.7–51.9	[55]
			MCF-7	51.8	
Cytotoxic lupeol ester study	_	Lupeol derivative	HepG2	12.74	[56]
			MCF-7	9.4	
			HT-29	6.85	

**Table 2:** Physicochemical features of α-amyrin and β-amyrin, lupeol, and erythrodiol.

	MW	MR	LogP	n-HBA	n-HBD	Violations Number
						Lipinski
Rule	<500 D.a	<140 A^2^	≤5	<10	<5	≤2
C/1	426.72	134.88	4.63	1	1	Yes
C/2	426.72	135.14	4.80	1	1	Yes
C/3	426.72	135.14	4.72	1	1	Yes
C/4	442.72	136.04	4.34	2	2	Yes

**Table 3:** Pharmacokinetic features of α-amyrin, β-amyrin, lupeol, and erythrodiol.

Models	ADME and toxicity properties													
	Absorption	Distribution		Metabolism							Excretion	Toxicity		
	Intestinal absorption (human)	BBB permeability	CNS permeability	CYP450							Total clearance	AMES toxicity	Hepatotoxicity	Skin sensitization
				Substrate		Inhibitor								
				2D6	3A4	1A2	2C19	2C9	2D6	3A4				
Unity	Numeric (%absorbed)	Numeric (Log BB)	Numeric (Log PS)	Categorical (YES/NO)							Numeric (log mL min^−1^ kg^−1^)	Categorical (yes/no)		
Predicted values														
C/1	96.716	0.69	−1.576	No	Yes	No	No	No	No	No	−0.044	No	No	No
C/2	97.241	0.695	−1.589	No	Yes	No	No	No	No	No	0.119	No	No	No
C/3	99.868	0.739	−1.35	No	Yes	No	No	No	No	No	0.153	No	No	No
C/4	97.028	−0.09	−1.291	No	Yes	No	No	No	No	No	0.043	No	Yes	No

**Table 4:** Binding energies in kcal/mol for the major compounds and docked co-crystallized ligands in complex with the targeted receptors.

Binding Energy	α-Amyrin	β-Amyrin	Lupeol	Erythrodiol	Co-Crystallized Ligand
6O0K protein	−9.51	−9.97	−9.78	−9.21	−8.15
3ERT protein	−9.96	−10.08	−10.17	−9.94	−7.21
6SJA protein	−10.86	−9.78	−8.15	−8.77	−8.75

**Table 5:** Grid parameters used for molecular docking.

Protein	Grid Spacing (Å)	Grid Centre Coordinates (X, Y, Z)	Grid Dimensions
6O0K	0.375 Å	−3.591, 1.798, −11.441	100 × 100 × 100
3ERT	0.375 Å	22.506, 5.5, 21.958	100 × 100 × 100
6SJA	0.375 Å	−7.731, 33.613, −11.723	100 × 100 × 100

## Data Availability

The original contributions presented in this study are included in the article. Further inquiries can be directed to the corresponding author.

## Supplementary Materials

The following supporting information can be downloaded at: https://www.mdpi.com/article/10.3390/molecules31173011/s1, Figure S1: The fragmentation mechanism peaks for α, β-amyrin; Figure S2: Chromatogram of fraction F2 showing integrated peaks and relative area percentages; Figure S3: The fragmentation mechanism peaks for lupeol; Figure S4: The fragmentation mechanism peaks for ψ-taraxasterol; Figure S5: The fragmentation mechanism peaks for taraxasterol; Figure S6: Chromatogram of fraction F4 showing integrated peaks and relative area percentages; Figure S7: The fragmentation mechanism peaks for erythrodiol; Figure S8: NMR-H1 spectra of erythrodiol; Figure S9: ^13^C-NMR spectral of erythrodiol; Figure S10: DEPT spectra of erythrodiol; Figure S11: Re-docking poses through superposition of the docked (in yellow) and native co-crystallized ligands within the active sites of 6O0K (A), 3ERT (B), and 6SJA (C) targeted receptors; Table S1: Relative peak area (%) distribution of compounds in fraction F2 based on TIC; Table S2: TIC-based purity assessment of fraction F4 corresponding to Peak 3.

## Abbreviations

The following abbreviations are used in this manuscript:

ADMET	Absorption, Distribution, Metabolism, Excretion, and Toxicity
ANOVA	Analysis of Variance
BAX	BCL-2-Associated X Protein
BBB	Blood–Brain Barrier
BCL-2	B-Cell Lymphoma 2
CNS	Central Nervous System
CYP450	Cytochrome P450
DCFH-DA	2′,7′-Dichlorodihydrofluorescein Diacetate
DEPT	Distortionless Enhancement by Polarization Transfer
DMSO	Dimethyl Sulfoxide
EI	Electron Ionization
ERα	Estrogen Receptor Alpha
FITC	Fluorescein Isothiocyanate
FSC	Forward Scatter
FSC-A	Forward Scatter Area
FSC-H	Forward Scatter Height
GC–MS	Gas Chromatography–Mass Spectrometry
HBA	Hydrogen-Bond Acceptor
HBD	Hydrogen-Bond Donor
HIA	Human Intestinal Absorption
HPV16	Human Papillomavirus Type 16
IC_50_	Half-Maximal Inhibitory Concentration
IRF3	Interferon Regulatory Factor 3
JC-1	5,5′,6,6′-Tetrachloro-1,1′,3,3′-Tetraethylbenzimidazolylcarbocyanine Iodide
JNK	c-Jun N-Terminal Kinase
MAPK	Mitogen-Activated Protein Kinase
MR	Molar Refractivity
MTT	3-(4,5-Dimethylthiazol-2-yl)-2,5-Diphenyltetrazolium Bromide
MW	Molecular Weight
NIST	National Institute of Standards and Technology
NMR	Nuclear Magnetic Resonance
PARP	Poly(ADP-Ribose) Polymerase
PBS	Phosphate-Buffered Saline
PDB	Protein Data Bank
PDBQT	Protein Data Bank, Partial Charge (Q), and Atom Type (T) Format
PI	Propidium Iodide
PI3K	Phosphoinositide 3-Kinase
RDA	Retro-Diels–Alder
RMSD	Root-Mean-Square Deviation
ROS	Reactive Oxygen Species
SD	Standard Deviation
SSC	Side Scatter
TLC	Thin-Layer Chromatography
TMS	Tetramethylsilane
TMRE	Tetramethylrhodamine Ethyl Ester
UV	Ultraviolet
