# Supplementary material for: Cytotoxic Triterpenes from Argan Pulp (Argania spinosa): Isolation, In Vitro Evaluation on Cancer Cell Lines, and In Silico Studies
Source: Molecules. 2026 Aug 27;31(17):3011. doi: 10.3390/molecules31173011 (PMC13567469; doi:10.3390/molecules31173011)
Supplement: Supplementary file 1 [file molecules-31-03011-s001.zip › molecules-4489798-supplementary.pdf]

SUPPLEMENTARY MATERIALS

Cytotoxic Triterpenes from Argan Pulp (*Argania spinosa*): Isolation, In Vitro Evaluation on Cancer Cell Lines, and In Silico Studies

Asma El Kaourat [1], Badr Eddine Kartah [1], Mohamed El Fadili [2], Noura Bentarhlia [1], Hamza Tachallait [3], Yassine Jaouhari [4,*], Matteo Bordiga [4], Zineb Ourradi [5], Younes Zaid [5], Zoubida Charrouf [1] and Hanae El Monfalouti [1]

[1] Laboratory of Plant Chemistry, Organic and Bioorganic Synthesis, Faculty of Sciences, Mohammed V
University in Rabat, Agdal, Rabat 10000, Morocco; asma.elkaourat@gmail.com (A.E.K.); b.kartah@um5r.ac.ma (B.E.K.); noura.bentarhlia@um5r.ac.ma (N.B.); zcharrouf@yahoo.fr (Z.C.);
h.elmonfalouti@um5r.ac.ma (H.E.M.)

[2] Laboratoire d'Ingénierie, Modélisation et Analyse des Systèmes (LIMAS Laboratory), Chemistry Department, Faculty of Sciences Dhar El Mahraz, Sidi Mohamed Ben Abdellah University,
Fez 30000, Morocco; mohamed.elfadili@usmba.ac.ma

[3] Department of Chemical and Biochemical Sciences—Green Process Engineering (CBS-GPE), Mohammed VI Polytechnic University (UM6P), Ben Guerir 43150, Morocco; hamza.tachallait@um6p.ma

[4] Department of Pharmaceutical Science, Università degli Studi del Piemonte Orientale "A. Avogadro", Largo Donegani 2, 28100 Novara, Italy; matteo.bordiga@uniupo.it

[5] Materials, Nanotechnologies and Environment Laboratory, Department of Biology, Faculty of Sciences, Mohammed V University in Rabat, Rabat 10000, Morocco; ourradizineb@gmail.com (Z.O.); y.zaid@um5r.ac.ma (Y.Z.)

* Correspondence: yassine.jaouhari@uniupo.it
Tel.: +39-(0321)-375875

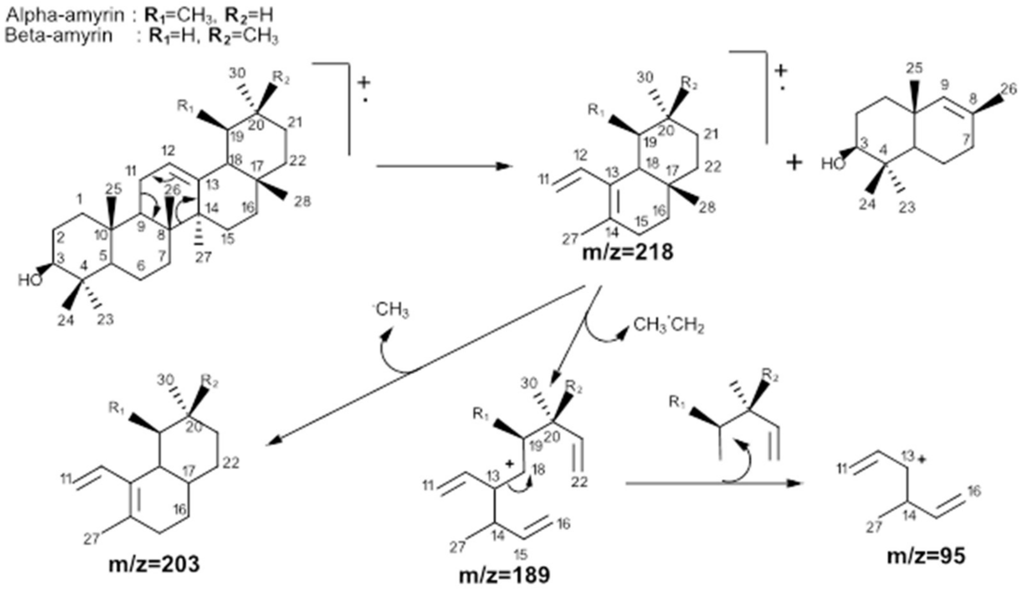

Figure S1: The fragmentation mechanism peaks for α, β-amyrin.

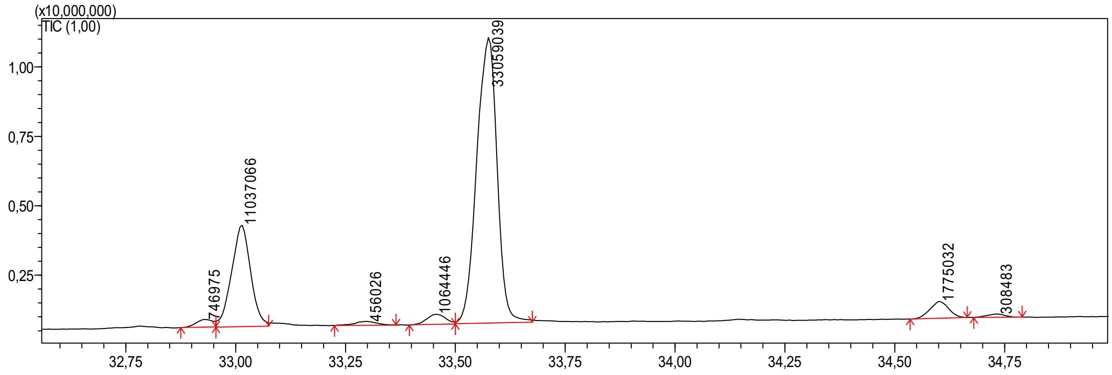

Figure S2: Chromatogram of fraction F2 showing integrated peaks and relative area percentages

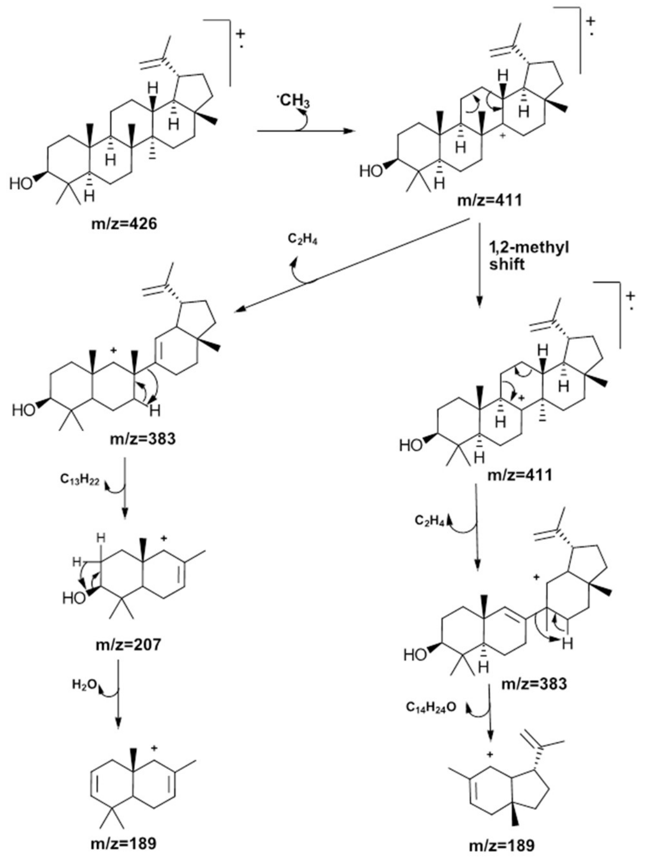

Figure S3: The fragmentation mechanism peaks for lupeol.

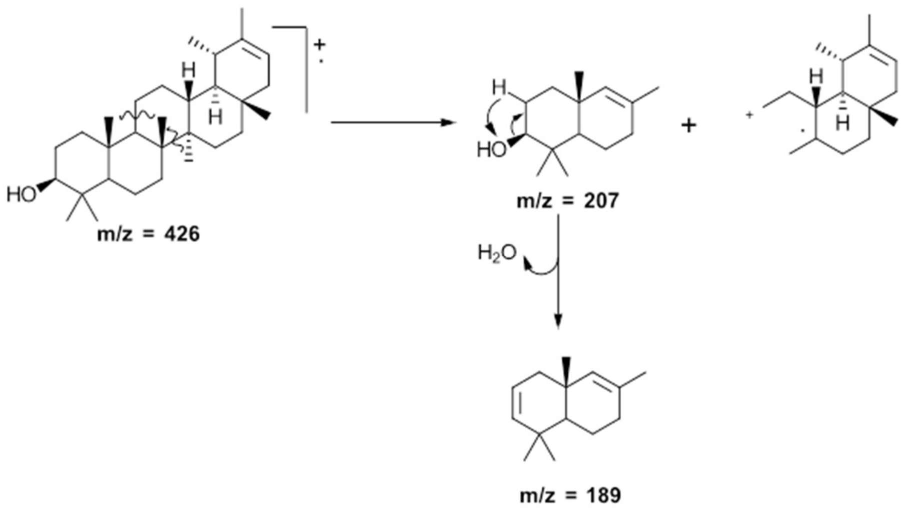

Figure S4: The fragmentation mechanism peaks for ψ-taraxasterol.

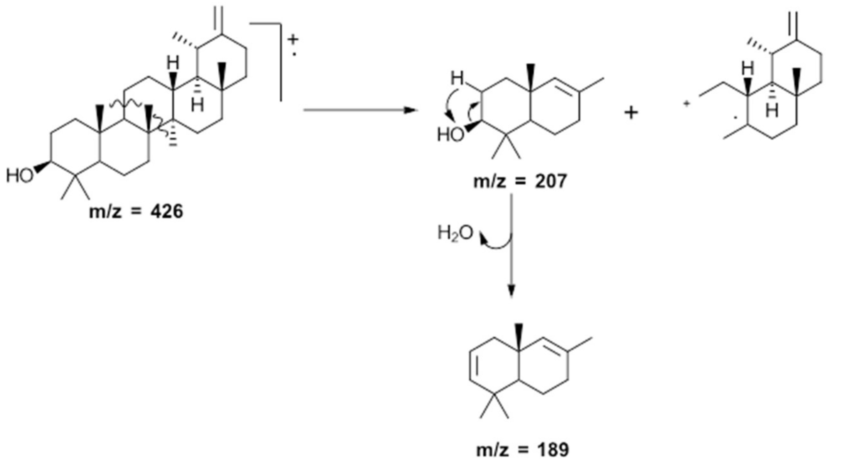

Figure S5: The fragmentation mechanism peaks for taraxasterol.

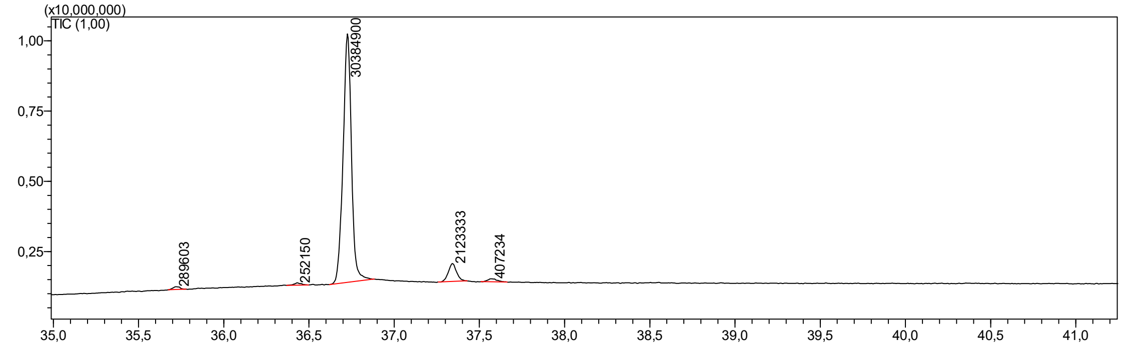

Figure S6: Chromatogram of fraction F4 showing integrated peaks and relative area percentages

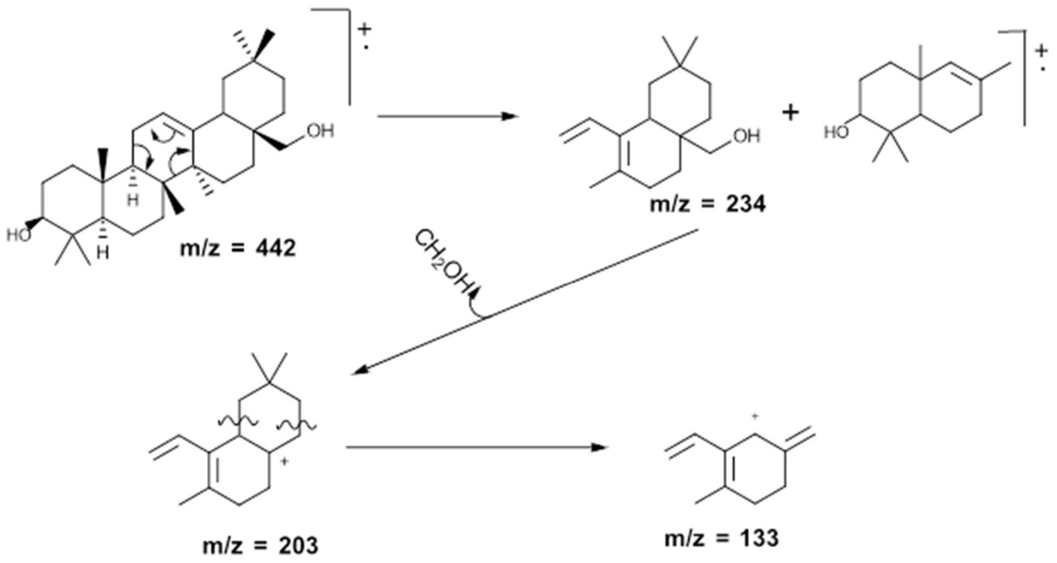

Figure S7: The fragmentation mechanism peaks for erythrodiol.

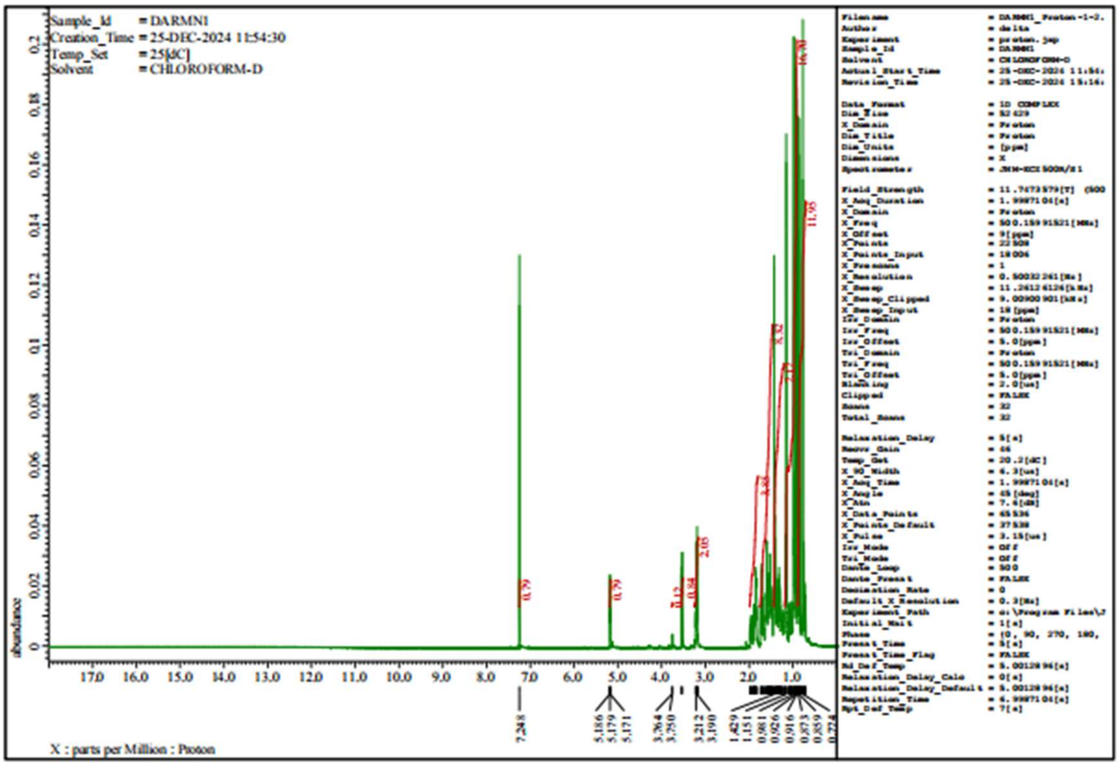

Figure S8: NMR-H1 spectra of erythrodiol

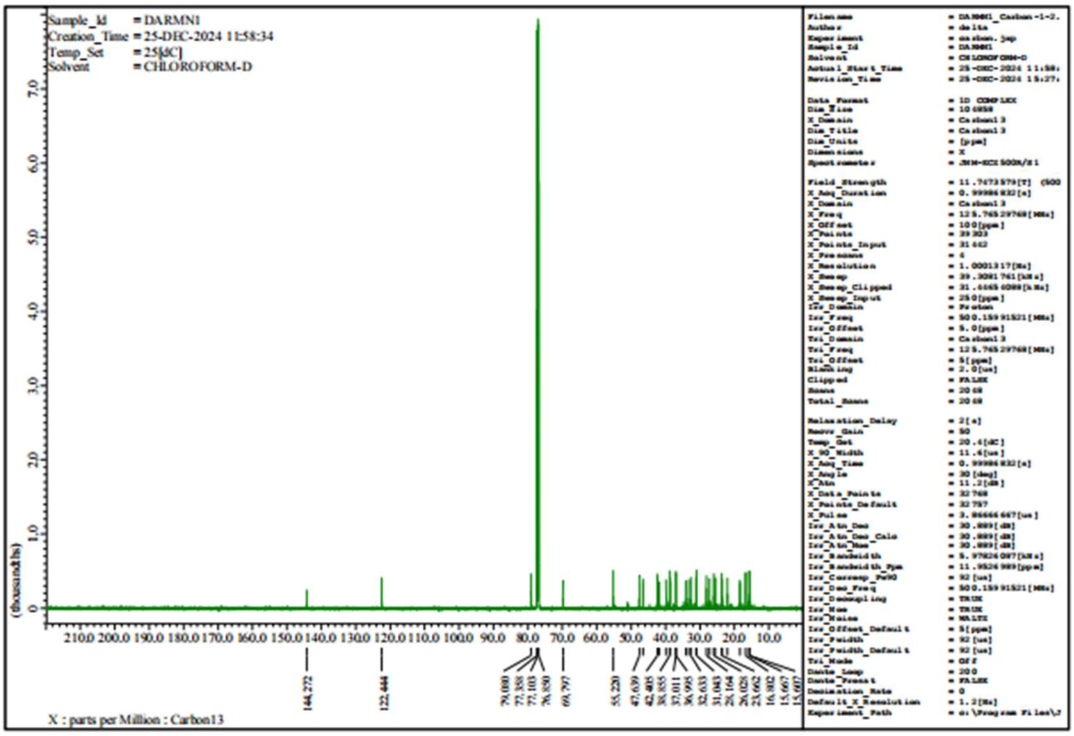

Figure S9: ^{13}C-NMR spectral of erythrodiol

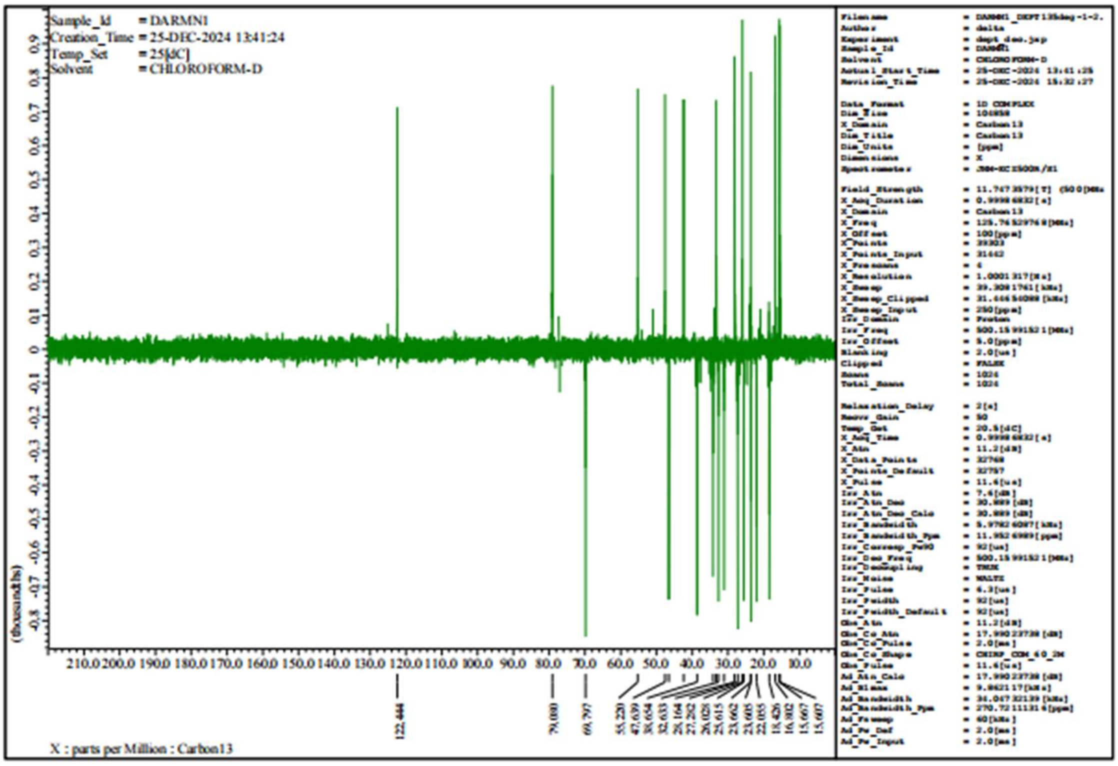

Figure S10: DEPT spectra of erythrodiol

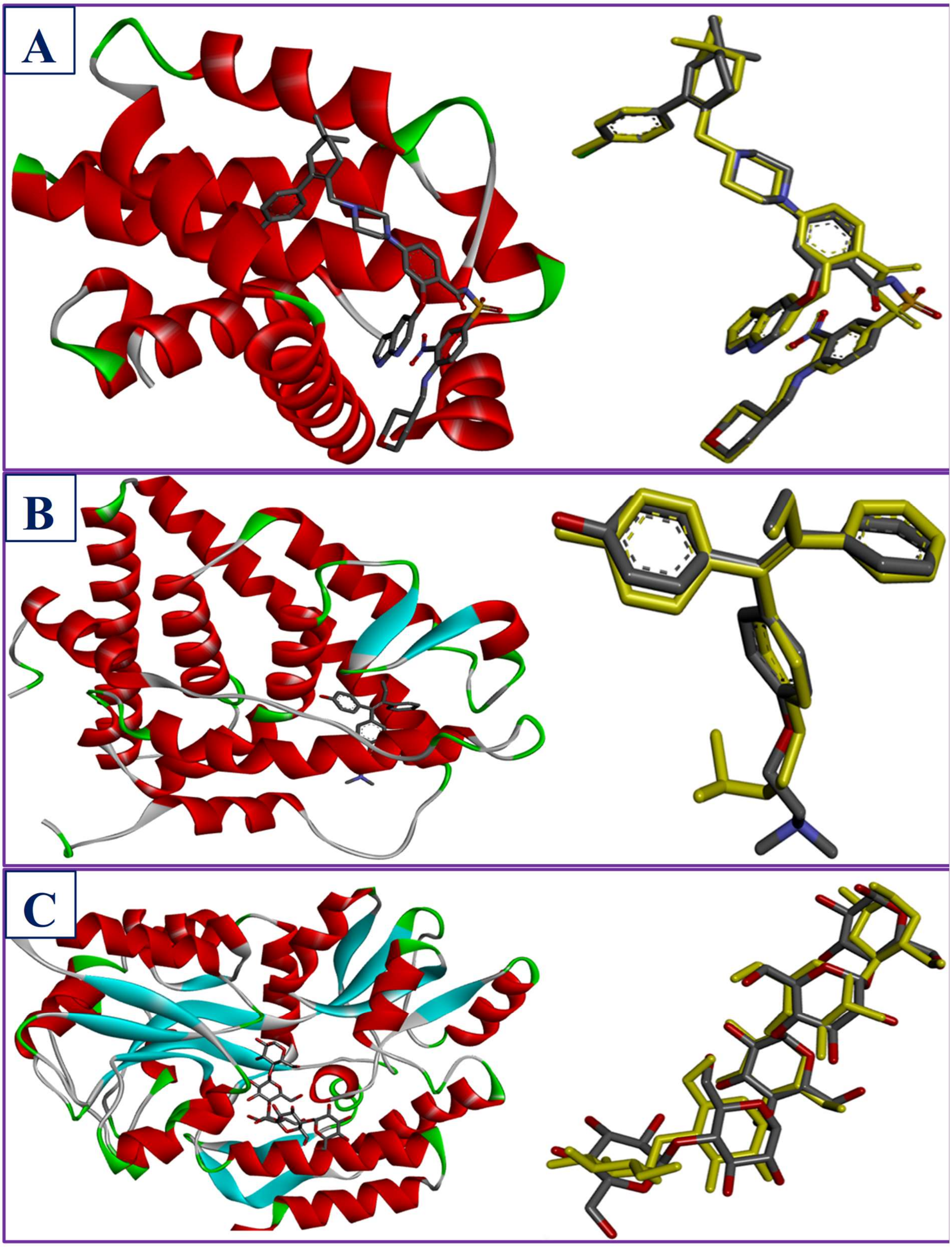

Figure S11: Re-docking poses through superposition of the docked (in yellow) and native co-crystallized ligands within the active sites of 6O0K (A), 3ERT (B), and 6SJA (C) targeted receptors.

Table S1: Relative peak area (%) distribution of compounds in fraction F2 based on TIC.

Peak #	Assigned peak	Retention time (min)	Start time (min)	End time (min)	m/z	Area	Area (%)	Height	Height (%)	A/H	Mark
1	—	32.930	32.870	32.955	TIC	74,675	1.54	273,795	1.78	2.73	MI
2	Peak A	33.013	32.955	33.075	TIC	1,107,066	22.78	3,646,381	23.68	3.03	MI
3	—	33.298	33.220	33.365	TIC	45,026	0.94	133,337	0.87	3.42	MI
4	—	33.456	33.395	33.500	TIC	106,446	2.20	353,812	2.30	3.01	MI
5	Peak B	33.575	33.500	33.675	TIC	3,305,903	68.24	10,280,266	66.77	3.22	MI
6	Peak C	34.602	34.535	34.665	TIC	177,503	3.66	597,214	3.88	2.97	MI
7	Peak D	34.734	34.680	34.790	TIC	30,849	0.64	111,554	0.72	2.77	MI

TIC: total ion chromatogram; A/H: area-to-height ratio; MI: manually integrated peak. Peaks A–D correspond to the main chromatographic components selected within fraction F2. Relative abundance was evaluated using the TIC peak area percentage

Table S2: TIC-based purity assessment of fraction F4 corresponding to Peak 3

Peak #	Retention time (min)	Start time (min)	End time (min)	m/z	Area	Area (%)	Height	Height (%)	A/H	Mark
1	35.721	35.675	35.775	TIC	28,603	0.87	100,463	1.03	2.88	MI
2	36.431	36.370	36.495	TIC	25,210	0.75	84,971	0.87	2.97	MI
3	36.720	36.620	36.880	TIC	3,038,490	90.82	8,845,724	90.50	3.43	MI
4	37.341	37.260	37.425	TIC	212,333	6.35	630,091	6.51	3.34	MI
5	37.573	37.510	37.660	TIC	40,734	1.22	107,420	1.10	3.79	MI

TIC: total ion chromatogram; A/H: area-to-height ratio; MI: manually integrated peak. The target fraction F4 corresponded to Peak 3, which represented 90.82% of the total TIC area.
